# Trading off stability against activity in extremophilic aldolases

**DOI:** 10.1038/srep17908

**Published:** 2016-01-19

**Authors:** Markus Dick, Oliver H. Weiergräber, Thomas Classen, Carolin Bisterfeld, Julia Bramski, Holger Gohlke, Jörg Pietruszka

**Affiliations:** 1 Institute of Bioorganic Chemistry, Heinrich-Heine-Universität Düsseldorf im Forschungszentrum Jülich, and Bioeconomy Science Center (BioSC), Jülich, Germany; 2 Institute of Complex Systems ICS-6: Structural Biochemistry, Forschungszentrum Jülich GmbH, Jülich, Germany; 3 Institute of Bio- and Geosciences IBG-1: Biotechnology, Forschungszentrum Jülich GmbH, Jülich, Germany; 4 Institute of Pharmaceutical and Medicinal Chemistry, Heinrich-Heine-Universität Düsseldorf, Düsseldorf, Germany

## Abstract

Understanding enzyme stability and activity in extremophilic organisms is of great biotechnological interest, but many questions are still unsolved. Using 2-deoxy-D-ribose-5-phosphate aldolase (DERA) as model enzyme, we have evaluated structural and functional characteristics of different orthologs from psychrophilic, mesophilic and hyperthermophilic organisms. We present the first crystal structures of psychrophilic DERAs, revealing a dimeric organization resembling their mesophilic but not their thermophilic counterparts. Conversion into monomeric proteins showed that the native dimer interface contributes to stability only in the hyperthermophilic enzymes. Nevertheless, introduction of a disulfide bridge in the interface of a psychrophilic DERA did confer increased thermostability, suggesting a strategy for rational design of more durable enzyme variants. Constraint network analysis revealed particularly sparse interactions between the substrate pocket and its surrounding α-helices in psychrophilic DERAs, which indicates that a more flexible active center underlies their high turnover numbers.

Enzymes from thermophilic organisms are of particular interest to biotechnology due to their adaptation to high temperatures^1^,^2^. As a result, considerable effort is being put into screening for new, thermophilic enzyme variants. An alternative route is to re-design proteins from mesophilic organisms, increasing their thermostability. However, rational identification of hotspots affecting enzyme stability is still a challenging task and is based on sophisticated knowledge on structural determinants responsible for thermostability. In recent years, many new protein structures from meso- and thermophilic organisms have been determined, and large genome libraries from a variety of species have been compiled^3^. Aside from well-known stabilizing features such as hydrophobic cores, disulfide and salt bridges^4^, enzymes can be stabilized by strengthening intermolecular interactions in oligomeric structures^5^,^6^. However, compared to other stabilizing factors, the properties of oligomeric interfaces have received minor attention in studies dealing with adaption to high temperature.

Thermal adaptation has also evolved to low temperatures. Psychrophilic organisms have developed many modulations on a cellular and molecular level to survive under those extreme conditions. While the high catalytic activity and broad substrate spectrum of most psychrophilic enzymes make them attractive tools for biotechnology^7^,^8^,^9^, their industrial application is hampered by their short life-times under typical fermentation conditions. Structural characteristics of these proteins include clusters of glycine residues and a reduced number of charged and hydrophobic side chains^10^. However, due to the low number of available 3D structures (<100 Protein Data Base-PDB-entries containing ”psychrophilic” or ”cold-adapted” in October 2015), aspects of the structural origin of low-temperature adaption are awaiting further investigation.

The 2-deoxy-D-ribose-5-phosphate aldolase (DERA) is an enzyme involved in nucleotide catabolism^11^. As it is found in all kingdoms of life, including psychrophilic and hyperthermophilic organisms, it represents a suitable model system for studying adaptation to extreme temperatures. The enzyme catalyzes the formation of C-C bonds between an aldehyde as an electrophile and acetaldehyde as a nucleophile in a highly stereoselective manner^12^. Thus, it has become an important alternative to chemical methods to synthesize chiral building blocks for natural products^13^,^14^. During the past decade, four crystal structures of hyperthermophilic DERAs were solved^15^,^16^,^17^, but thus far no structure of psychrophilic origin is available.

Here we present comparative biochemical, structural, and computational studies of DERAs from psychrophilic, mesophilic and thermophilic organisms. In order to establish a solid foundation for further experiments, we have determined the first crystal structures of DERAs derived from cold-adapted organisms. Properties affecting the stability against thermal inactivation are investigated with a deliberate focus on the role of the dimer interface. Finally, we show likely reasons for the very different biophysical characteristics of DERAs from psychrophilic vs. mesophilic organisms.

## Results

### Biochemical characterization

**Figure 1:**
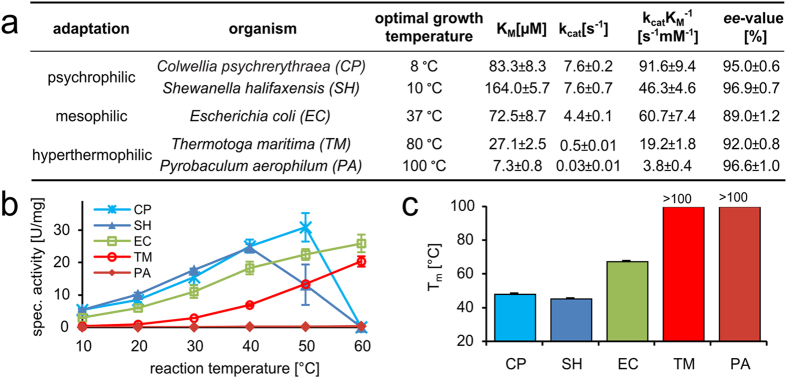
Kinetic and biophysical properties of DERAs used in this study. (**a**) Overview of all used DERA orthologs with optimal growth conditions of their hosts^57^,^58^,^59^,^60^, Michaelis-Menten parameters obtained from steady-state kinetics and enantiomeric excess (*ee*) of the conversion of propanal and acetaldehyde to (*R*)-3-hydroxy-pentanal. (**b**) Specific activity at different reaction temperatures. (**c**) Melting temperatures have been obtained by CD spectroscopy at 222 nm. All error bars represent the standard deviation.

Different DERA orthologs from psychrophilic (*Colwellia psychrerythraea*, *Shewanella halifaxensis*), mesophilic (*Escherichia coli*) and hyperthermophilic (*Pyrobaculum aerophilum*, *Thermotoga maritima*) organisms were used for this study (Fig. 1a). DERAs from psychrophilic organisms have not been characterized until now. After cloning, expression and purification, pure (>95%) and active enzymes from all organisms were obtained. Initially, biochemical and biophysical characteristics of all five DERA orthologs were compared (Fig. 1). The DERA-catalyzed aldol reaction between acetaldehyde and propanal to 3-hydroxy-pentanal showed a high enantiomeric excess (89–96%) for the (*R*)-enantiomer in all cases. Clear differences in terms of activity and stability were determined. Both K_M_ and k_cat_ values decrease with increasing optimal growth temperature of the organism (details on the Michaelis-Menten data are provided as Supplementary Fig. S1a–c).

For all enzymes specific activity shows a steady increase with temperature (Fig. 1b), until the melting-range (Fig. 1c) is reached. This is at variance with previous reports for enzymes from psychrophilic organisms, where activity was shown to already drop at lower temperatures^9^. Due to the short reaction time of 1 min, time-dependent denaturation is delayed in this assay. This explains why DERA_CP_ displays a temperature optimum above its melting point. Between 10 and 60 °C, DERA_PA_ does not exhibit a specific activity higher than 1.5% that of DERA_EC_. Overall, our biochemical data of DERA_EC_, DERA_TM_ and DERA_PA_ are in agreement with those from Sakuraba *et al*.^17^. Stabilities of all DERA orthologs were determined with both CD spectroscopy (Fig. 1c, an example spectrum for *T*_m_ determination is shown in Supplementary Fig. S1d,e) and an activity-based assay (Supplementary Fig. S1f). DERAs from psychrophilic species had a similar thermostability and were around 20 K less stable than DERA_EC_. No melting point could be identified for hyperthermophilic DERAs, thus it must exceed 100 °C in both cases. However, when incubated at 100 °C, only DERA_PA_ was stable over night at that temperature, while DERA_TM_ had a half-life of 22 min. These observations prompted us to investigate structural reasons for the diverse properties found with DERAs of different thermal adaptation.

### Crystallization of psychrophilic DERAs

As no structure was available for any psychrophilic DERA, crystallization trials were performed, and protein crystals suitable for diffraction experiments were obtained. The X-ray structures of both psychrophilic DERAs were determined with resolutions of 2.1 Å for *C. psychrerythraea* and 1.8 Å for *S. halifaxensis* (Supplementary Table S1). Superposition of the structures with those of meso- and hyperthermophilic DERAs revealed a high similarity of the monomeric units, with root-mean-square (r.m.s.) deviations (RMSD) in the range of 0.63–1.65 Å (Supplementary Fig. S2). In view of this high structural similarity, it is interesting to note that the sequence identity between hyperthermophilic and meso-/psychrophilic DERAs is quite low (29–31%, see Supplementary Fig. S2). Nevertheless, the conservation of all catalytically important residues (e.g., K167 and D102, numbering according to *E. coli* DERA) together with a virtually identical fold strongly suggests a common evolutionary origin of all five DERA orthologs. The structures display a canonical TIM barrel fold built up of eight β-α repeats in a toroidal arrangement, with an additional N-terminal helix (α0) directly adjacent to the C-terminal helix α8 (vide infra). Furthermore, all DERAs investigated in this study form constitutive dimers. We note that the relative orientations of protomers as well as the characteristics of the dimer interface for both psychrophilic DERAs closely resemble the situation in the mesophilic (*E. coli*) enzyme. In contrast, hyperthermophilic aldolases have evolved a different dimeric arrangement (discussed below). Comparing the substrate binding pockets of the new structures with DERA_EC_, no amino acid exchanges or significant conformational differences were found within a radius of 5 Å about the natural substrate (as represented by the covalent intermediate included in PDBID 1JCL^12^). With the 3D structures of all five DERAs at hand, sophisticated structure-based studies such as rational enzyme design and computational investigations could be performed.

### Structural features related to thermostability

**Figure 2:**
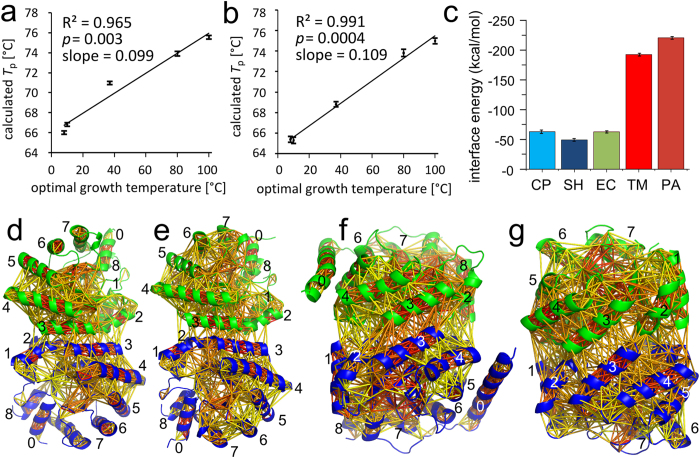
Structural rigidity of DERAs from psychrophilic, mesophilic, and hyperthermophilic organisms, as calculated by CNA. (**a,b**) Correlation of phase transition temperature *T*_p_ computed by CNA over ensembles from MD simulations in the range of (**a**) 5–50 ns and (**b**) 50–100 ns with the optimal growth temperature of the host. Error bars represent the standard error of the mean. (**c**) Sum over energies of all rigid contacts between residue pairs at the dimer interface. (**d–g**) Visualization of rigid contact strength between amino acid pairs for (**d**) DERA_CP_ (DERA_SH_ showed a pattern that was nearly identical), (**e**) DERA_EC_, (**f**) DERA_TM_, and (**g**) DERA_PA_. Different orientations of the structures result from the non-equivalent dimer arrangements in psychro-/mesophilic vs. hyperthermophilic DERAs. The strength of the interaction is color coded from yellow (weak) to red (strong).

To identify the structural properties underlying the stabilization effects in hyperthermophilic DERAs and the increased catalytic efficiency of psychrophilic DERAs, we set out to quantify inter- and intramolecular interactions by *in silico* methods (PDB IDs used for computational studies are given in Supplementary Table S2). Our initial strategy was to determine the number and strength of non-covalent interactions (hydrogen bonds, salt bridges, and hydrophobic contacts) in the crystal structures using the FIRST software^18^, which did not give a satisfying result as there was no correlation between the calculated number of hydrogen bonds/salt bridges and measures of thermostability (Supplementary Table S3). For hydrophobic interactions, an R^2^ of 0.91 was determined, suggesting a correlation between thermostability and the number of hydrophobic contacts. However, the number of hydrophobic contacts does not allow differentiating between DERA_SH_ and DERA_EC_, or between the two hyperthermophilic DERAs, although these two proteins clearly differ in thermostability. The quantification of non-covalent interactions in ensembles of conformations generated by 3 × 50 ns molecular dynamics (MD) simulations could partly improve R^2^ in the case of hydrogen bonds and salt bridges (Supplementary Table S3), but good correlations (R^2^ ≥ 0.8) were only obtained if the strongest and least frequent hydrogen bonds and salt bridges were considered. Furthermore, now the number of hydrophobic contacts does not correlate with thermostability (R^2^ = 0.08). Overall, this outcome shows that purely counting non-covalent interactions yields correlations with thermostability. However, the quality strongly depends on the structural origin and the particular choice of descriptor. Hence, we hypothesized that, in addition to the frequency, the distribution of the interactions within the protein is important. This hypothesis was probed by constraint network analysis (CNA) recently developed by Pfleger *et al*.^19^. Here, a protein is represented as a network of constraints deduced from covalent and non-covalent interactions, from which information on the biomacromolecular flexibility/rigidity is computed^20^. In CNA, thermal unfolding of a protein is simulated by gradually removing hydrogen bond constraints from the network in the order of increasing strengths^21^. It has been shown that the temperature of the resulting phase transition *T*_p_ from a largely rigid to a largely flexible network (which can be also described as a melting point) correlates with measures of thermostability, if proteins from the same structural family are compared^21^,^22^. For all five DERA orthologs, three independent MD simulations of 100 ns each were performed. Evaluation of these MD simulations via calculation of the RMSD of C_α_ atoms with respect to the corresponding starting structure (Supplementary Fig. S3a) and of radii of gyration (Supplementary Fig. S3b) showed that during the simulation time of 100 ns, all protein orthologs remained structurally stable (RMSD mostly <3.0 Å and standard deviation of the radius of gyration <2 Å). Furthermore, the r.m.s. inner product (RMSIP) over the first ten principal components^23^ shows for respective pairs of independent MD simulations of one ortholog that the conformational subspaces covered by the two MD simulations are similar to highly similar (range of RMSIP values: 0.63 to 0.79; Supplementary Table S4). From these simulations, initially, 1125 conformations in the time range from 5–50 ns were selected, and the *T*_p_-value for each snapshot was calculated via CNA. The frequencies of *T*_p_-values in the ensemble follow a Gaussian distribution in all cases (Supplementary Fig. S4). Note that the calculated *T*_p_-values should be considered relative values because the absolute phase transition temperatures may depend on the architecture and the size of the enzyme^19^. To see how robust the CNA results are with respect to the chosen structural ensemble, CNA calculations were repeated for the range of 50–100 ns. To validate the CNA results at the macroscopic level, experimental melting temperatures would be the best measures of thermostability. Unfortunately, a well-defined melting temperature is not available for the hyperthermophilic DERAs (>100 °C, see Fig. 1c). Thus, we instead used the optimal growth temperature of the source organisms (*T*_org_), which has been previously applied to compare meso- and thermophilic proteins^21^,^22^,^24^. *T*_org_ is an approximate descriptor for protein thermostability as, on average, the value for *T*_m_ of a protein is around 24 °C higher than the *T*_org_ of the corresponding species^24^. For evaluation, conformations of all three MD simulations were extracted, and the overall average and standard error of the mean of *T*_p_ were calculated for each DERA ortholog (Supplementary Table S5). The computed *T*_p_ shows a significant (p < 0.01) and very good correlation with *T*_org_ (R^2^ = 0.965 (0.991)) over the MD ensemble in the range of 5–50 ns (50–100 ns) (Fig. 2a,b). The range of predicted *T*_p_ values is smaller than that of experimental *T*_org_, in line with slopes of the correlation lines of ≈0.1. A reason for this may be that the *T*_p_ values rely on an empirical relationship introduced previously^21^,^25^, which has been determined for a dataset of orthologous meso- and thermophilic protein pairs with *T*_org_ in the range of 30–83 °C. The deviation indicates that a system-specific reparametrization might be necessary here. The values obtained from individual MD simulations, together with the standard errors of their means, are given in Supplementary Table S5. These results show that the *T*_p_ predictions from the individual MD simulations are consistent (maximal spread in *T*_p_ values across three trajectories: 3 °C), in line with the above RMSIP analysis. The differences between the computed *T*_p_ values for the first and second 50 ns are ≤2.2 K in all cases, strongly indicating converged calculations. The results suggest that the model underlying CNA correctly captures differences in the thermostability of the DERAs.

We next analyzed on a per-residue basis how changes in thermostability relate to changes in local structural stability (rigidity). The local structural stability in terms of the strength of rigid contacts was characterized, which describes when two residues cease to reside within one rigid region of the protein during the thermal unfolding simulation^25^,^26^. In Fig. 2d–g, the networks of rigid contacts are compared for different DERAs. Qualitatively, the two enzymes from hyperthermophilic organisms have very dense networks that also extend markedly across the dimerization interface. In contrast, DERAs from psychrophilic and mesophilic organisms show much fewer rigid contacts in the interface region. Furthermore, the DERAs in this group differ with respect to the rigid contacts between the outer helices 5–7 and the inner part of the TIM barrel (Fig. 2d,e). To quantify the influence of structural stability in the interface region on the thermostability of the DERAs, we computed the sum over energies of all rigid contacts between residue pairs at the dimer interface (Fig. 2c). The more negative this value, the more stable is the interface region. The results indicate that the interface regions of DERA_TM_ and DERA_PA_ are much more structurally stable than those of their mesophilic and psychrophilic counterparts.

### Design of monomeric DERAs

**Figure 3:**
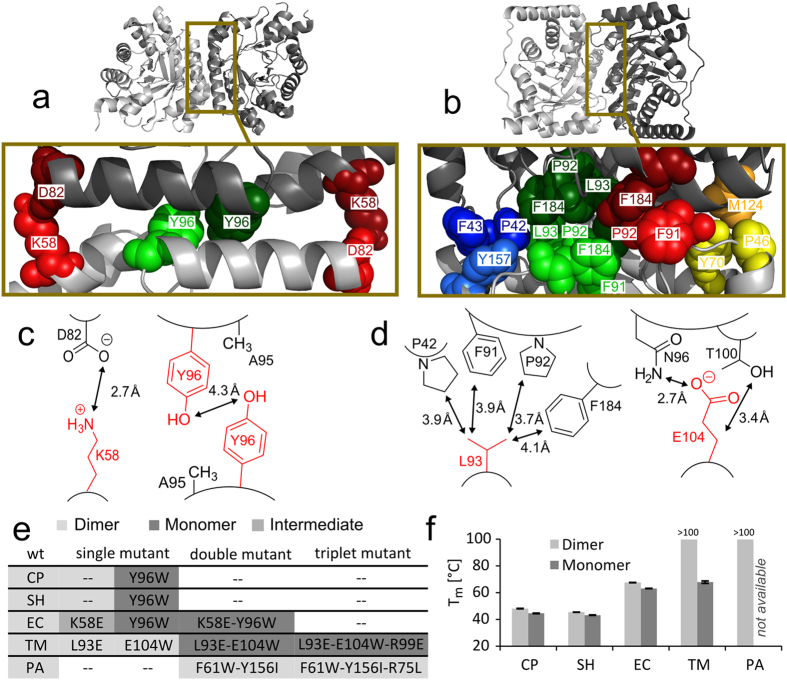
Design of mutants in the dimer interface of DERA orthologs. (**a,b**) Interactions in the dimer interface of (**a**) DERA_EC_ and (**b**) DERA_TM_. Amino acids belonging to the same pair or cluster are shown in CPK mode with similar colors. (**c,d**) Schematic representation of regions in (**c**) DERA_EC_ and (**d**) DERA_TM_ that were selected for modification. Mutated amino acids are labeled in red. (**e**) Overview of all designed mutants and their oligomeric states. Positionally equivalent sites are listed in the same column. (**f**) Stability of dimeric and monomeric DERAs from different organisms. Melting temperatures (with standard deviation) have been obtained by CD spectroscopy at 222 nm.

By comparing the structures of DERA_EC_, DERA_TM_ and DERA_PA_, Sakuraba *et al*. proposed that hydrophobic clusters within the dimer interface (Fig. 3a,b) strongly contribute to the overall thermostability of hyperthermophilic DERAs^17^. Our CNA results support the hypothesis of stronger interactions in the dimer interfaces of the thermostable DERAs. In order to verify these finding with *in vitro* experiments, DERA mutants were designed with the intention to weaken the affinity between monomeric subunits. Two different strategies were followed: Either a charged or hydrophobic interaction partner was mutated to generate a repulsive interaction (e.g., by a K58E mutation, which should repel D82), or a sterically demanding group such as a tryptophan was introduced, disturbing shape complementarity at the interface (Fig. 3c,d). Size exclusion chromatography was used to analyze the hydrodynamic sizes of the mutant proteins (an example is shown in Supplementary Fig. S5). Soluble and active monomeric variants for DERAs from *C. psychrerythraea*, *S. halifaxensis*, *E. coli* and *T. maritima* have been created following this methodology; specific mutations are denoted in Fig. 3e, together with the resulting oligomeric state. For the K58E-mutant of DERA_EC_, the hydrodynamic size was in between the values of the monomer and the dimer. Thus, it might feature a dynamic equilibrium between both states. Strikingly, DERA from *P. aerophilum* retained its original oligomeric state even after introducing a triple mutation. It is important to note that for the monomeric variants the specific activity did not change significantly (Supplementary Table S6). This preservation of function is a good indication that the overall structure of the protein has not been affected by altering the oligomeric state. Furthermore, the *T*_m_ values of all monomerized DERAs with either psychrophilic or mesophilic origin decreased by only a few Kelvin (Fig. 3f). The same holds true for the activity half-life at elevated temperature (Supplementary Table S6). In contrast, conversion of the hyperthermophilic DERA from *T. maritima* to a monomer decreased its stability by more than 30 K. These results provide experimental support for the hypothesis of Sakuraba and co-workers^17^. While dimerization via the native interface is not important for the stability of meso- and psychrophilic DERAs, the much more extensive interactions in the *T. maritima* variant appear to be an essential determinant of thermal stability.

### Stabilizing the dimer interface

**Figure 4:**
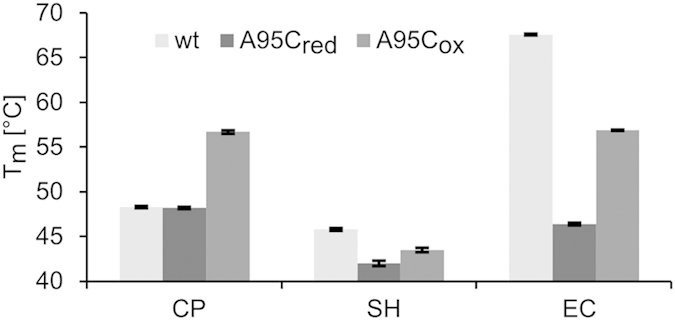
Melting points of oxidized and reduced forms of A95C mutants vs. wt enzymes. The numbering corresponds to DERA_EC_. Melting temperatures have been obtained by CD spectroscopy at 222 nm. Error bars represent the standard deviation.

We were able to destabilize a hyperthermophilic DERA by disturbing the interaction network between the monomeric subunits. For use in biotechnology, however, the converse approach, i.e., ways of stabilizing enzymes, is of particular interest. Hence, the finding that strong intermolecular interactions may promote thermostability inspired an attempt to enhance the interfacial interaction of DERAs from *C. psychrerythraea*, *S. halifaxensis*, and *E. coli*. Introducing a novel hydrophobic cluster similar to those found in both hyperthermophilic DERAs is a possible strategy. However, this approach would imply extensive mutations increasing the risk of negative side effects (influence on expression level, specific activity, and stability). Therefore, an artificial disulfide bridge was introduced because this is the only covalent (except for rare isopeptide bonds) and thus the strongest interaction between two non-neighboring residues in a protein. According to the empirical rules proposed by Hazed *et al*.^27^, the distances between C_β_-atoms of both protein chains were determined, and all pairs (excluding prolines) with a distance of 3.83 ± 0.18 Å were selected. For all three non-hyperthermophilic DERAs one pair was found: A95–A95. As this position is located close to the C2 axis, only one mutation is necessary to provide both cysteines for the new disulfide bridge. After expression and purification of the A95C mutants, formation of the disulfide bridge was verified via SDS-PAGE under non-reducing conditions (only the oxidized mutant led to a 56 kDa band on the gel). The stability of all variants was tested by CD spectroscopy (Fig. 4). In the reduced state (i.e., without formation of the disulfide bridge), the A95C mutation turned out to negatively affect the stability of DERA_SH_ and DERA_EC_, but did not influence the stability of DERA_CP_. It remains unclear why in two out of three cases the A95C mutation has a negative effect on thermostability. However, these results are not surprising as studies have shown that more than 80% of all single point mutations in a protein decrease its stability^28^. Importantly, oxidation of the Cys residues to the disulfide bridge results in an increased thermostability in the case of all three proteins. While in DERA_SH_ and DERA_EC_ the melting point of the oxidized form was still lower than the wt value, the exchange led to a marked increase of 8.4 K in thermostability in the case of DERA_CP_. Further tests for the DERA_CP_-A95C_ox_ variant revealed a much longer half-life at 50 °C (>80 min vs. 1 min for the wt) and 86 ± 8% of the wt activity. Overall, the results demonstrate that it is possible to increase the thermal stability of a non-thermophilic DERA by strengthening the interactions at the dimer interface.

### Features promoting activity in psychrophilic DERAs

**Figure 5:**
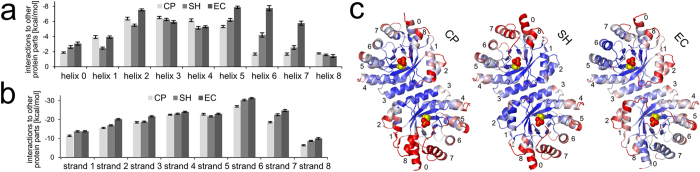
Comparison of structural stability and mobility in secondary structure elements of psychro- and mesophilic DERAs. (**a,b**) Sum over energies of rigid contacts between residues of each (**a**) α-helix and (**b**) β-strand of DERA_EC_, DERA_CP_, and DERA_SH_ and the rest of the protein without the respective secondary structure element as computed by CNA. The values are divided by the number of amino acids in the secondary structure element and thus represent an average value with standard error. (**c**) Relative B-factor distribution from blue (mean – SD) to red (mean + SD). The natural substrate is represented in CPK mode (phosphate group in red/orange, other atoms in yellow). For DERA_EC_, PDBID 1JCL^12^ has been used.

Our CNA data did not reveal major differences in the structural stabilities of the dimer interfaces of meso- and psychrophilic DERAs. Thus, experimental differences in thermostability and activity parameters (Fig. 1) most probably arise from differences in the type and strength of intramolecular interactions in these proteins. Along these lines, CNA suggested a varying density of rigid contacts between secondary structure elements (Fig. 2d–e). To further investigate this aspect, the sum over energies of rigid contacts connecting each α-helix or β-strand to the rest of the protein was computed (Fig. 5a,b). In all three DERAs, the β-strands are connected more strongly to other protein parts than the helices. Comparing DERA_EC_ with DERA_CP_ and DERA_SH_, no difference can be seen between corresponding β-strands. In contrast, helices 5 and, particularly, 6 and 7 show markedly reduced strengths of rigid contacts in the psychrophilic enzymes. These helices are placed directly opposite to the dimer interface (Fig. 5c), next to strands 6 and 7 containing the catalytically active K167 (Schiff base formation, numbering corresponds to DERA_EC_) and K201 (pK_a_ reduction of the active lysine), respectively.

This finding suggests a possible explanation why both DERAs from psychrophilic organisms have a higher catalytic activity than their mesophilic (and thermophilic) counterparts at comparable temperatures: A weaker connection to the outer helices implies a higher mobility of the adjacent β-α loops, which decorate the “catalytic face” of the TIM barrel fold. Since these loops contribute to both the substrate channel and the active center^29^,^30^, they are thought to modulate both catalytic specificity and rate of conversion. Indeed, mutations in the outer helices as well as the β-α loops have been previously demonstrated to increase the activity of a hyperthermophilic indole-3-glycerol phosphate synthase (TrpC) at low temperatures^31^. Kinetic data indicated that these mutations result in an increased rate of product release, presumably due to enhanced flexibility and reduced affinity of the phosphate binding site (which is also present in DERAs). Further support for this view is provided by X-ray crystallography. In structure refinement, B-factors are used to parameterize coordinate uncertainty, which is largely related to thermal motion on different scales. The relative B-factor distribution of the backbone atoms reveals a tendency towards increased flexibility in helices 0, 6, and 7 of the psychrophilic DERAs, compared to DERA_EC_ (Fig. 5c). A similar trend is observed for the respective β-α loops, particularly in the segments immediately preceding the helices. Hence, our X-ray structures provide independent clues in favor of the hypothesis that adaption to low temperatures in psychrophilic DERAs involves a more flexible environment of the substrate binding pocket.

## Discussion

In our studies we used 2-deoxy-D-ribose-5-phosphate aldolase (DERA) as a model enzyme to elucidate mechanisms of thermal adaptation of orthologs from psychrophilic (*C. psychrerythraea*, *S. halifaxensis*), mesophilic (*E. coli*) and hyperthermophilic (*T. maritima*, *P. aerophilum*) organisms. The biochemical characterization revealed two general trends: On the one hand, the catalytic efficiency decreases with increasing temperature of adaption while maintaining the enantioselectivity. On the other hand, the thermostability increases with optimal growth temperature of the source organism. Sakuraba *et al*.^17^ hypothesized that interactions in the dimeric interface play a major role in thermal adaptation of DERA_TM_ and DERA_PA_. We tested this assumption by creating mutants of all five DERAs resulting in formation of monomers rather than the (wildtype) dimers. While stability of DERAs from psychrophilic and mesophilic sources was not influenced significantly, stability of DERA from the thermophile *T. maritima* was severely reduced. This supports the hypothesis of the dimer interface as a stability determinant in thermostable proteins, but the concept does not extend to the native dimers of mesophilic and psychrophilic DERAs. Nevertheless, by installing a disulfide bridge in the interface of DERA_CP_ as a psychrophilic representative, the melting temperature could be increased. The result is in agreement with other studies conducted with unrelated proteins where either by *in vitro* evolution^32^ or by rational design^33^,^34^ thermal stability could be increased upon introduction of intermolecular disulfide bridges. Research in the Protein Data Bank showed that 61% of all protein entries indicate more than one chain in their biological units. Several reasons for protein oligomerization like regulation, reduction of genomic size and adaptation of particle concentration are known^35^. Most of these are directly related to the protein’s *in vivo* function. Enzymes in biotechnology are typically overexpressed for *in vitro* applications. Thus, as they still form oligomers, mutations to stabilize the interface can be utilized to enhance thermostability in mesophilic enzymes.

Adding to our mutational study, computational analyses by means of CNA revealed a differing extent of interface interactions. In the case of psychrophilic DERAs, no structural information was available prior to this study. The first two psychrophilic DERA structures presented here reveal that the inner sphere of the active site does not differ significantly from DERA_EC_. Hence, the reason for their increased catalytic efficiency must reside in the outer shell. CNA calculations as well as a B-factor analysis were indeed able to identify distinctive features. Specifically, patches of higher flexibility were detected in the area of β-α repeats 6 and 7, caused by weaker interactions between the sheath of helices and the inner β-barrel. This region is located opposite to the dimer interface and contains the catalytically important residues such as the active lysine and the pK_a_-lowering lysine. *In vitro* studies on the activity of enzymes with (β-α)_8_-barrel fold already suggested that the region surrounding the binding pocket influences the substrate conversion rate^31^,^36^. Our data support the notion that psychrophilic enzymes are structurally more flexible in their active sites, which is an important determinant of overall catalytic performance^10^,^37^.

In this study, MD simulations were primarily used for generating structural ensembles for *T*_p_ calculations by CNA. However, many other studies on cold-adapted enzymes included MD simulations and thus can be used to put our findings into perspective. Some of these studies showed that global characteristics such as a reduced number of hydrogen bonds^38^ and salt bridges^39^ contribute to a higher overall flexibility in certain psychrophilic enzymes. Our investigations on DERAs of different thermal adaptation did not clearly support this view. Other studies revealed that adaptation mostly occurs locally in specific regions: By comparing mesophilic and psychrophilic elastases, Papaleo *et al*. detected flexible loop regions around the binding pocket, which might cause a higher activity of the enzyme^40^,^41^, in accordance to what we found for DERAs. While most computational studies were performed with monomeric proteins, Papaleo *et al*. also analyzed intermolecular interactions of the dimeric, cold-adapted alkaline phosphatase, revealing a higher flexibility in the interface region, which might be directly related to enzyme reactivity^42^. Notably, for dimeric DERAs, such intermolecular contacts contribute to thermostability, with no influence on activity. In conclusion, intermolecular interactions in extremophilic enzymes can be used both to stabilize the protein at high temperatures and to enhance the catalytic activity in a cold environment and thus can play a key role in thermal adaptation.

When viewed in the broader context of protein evolution, the DERA structures investigated in this study reveal two fundamental strategies used to achieve adaptation to widely differing ambient temperatures. The psychrophilic/mesophilic regime does not require special measures to prevent thermal unfolding and is therefore compatible with a (stability-wise) ’neutral’ dimer interface. Nevertheless, dynamic properties of the catalytic center (and possibly other parts of the structure) still need to be adjusted in order to maintain biological activity within favorable bounds; this is achieved in a conservative manner via modulation of the density and strength of interactions between secondary structure elements. This strategy, however, is insufficient when extended into the high-temperature range. In addition to an overall increase in non-covalent interactions, (hyper-)thermophilic DERAs have thus adopted a peculiar dimer assembly with a strong stabilizing effect. A thorough comparison of DERA_TM_ and DERA_PA_ reveals an even tighter constraint network in the latter, in accordance with its higher working temperature, suggesting that adaptation of local stiffness is still relevant in the thermophilic group.

It is interesting to note that *T. maritima* and *P. aerophilum* belong to different kingdoms, the former being a bacterium while the latter is an archaeon. In view of this very distant relationship, the similarity in temperature adaptation of both DERAs (in particular regarding the dimer interface) is remarkable. Related to this observation, one can speculate that (a) this particular type of dimer has been present in a common ancestor of bacteria and archaea, or (b) a primordial gene has been exchanged at a later time by lateral transfer, followed by significant divergence of amino acid sequences, or (c) this particular strategy has emerged independently in the two phyla. While we cannot decide among these options based on our data, it is obvious that this adaptation must have been very stable on the evolutionary time scale.

In summary, this study provides new insights into both the stability of thermophilic (oligomeric) DERAs and the flexibility of their psychrophilic counterparts. In addition to the computational analyses, the concept of modifying the oligomeric state or interaction strength between protomers is important from an application point of view, such as stabilizing proteins for biotechnological applications.

## Methods

### Cloning, protein expression and purification

DERA-coding deoC-genes from *P. aerophilum*, *T. maritima* and *C. psychrerythraea* were synthesized by GenScript and cloned into the pET21a-vector with a C-terminal 6× His-Tag. The gene from *S. halifaxensis* was isolated from genomic DNA, and the deoC-gene from *E. coli* had been isolated previously^43^. Single point mutations were inserted via QuikChange (Stratagene) or round-the-horn^44^ mutagenesis with Phusion polymerase (list of primers is shown in Supplementary Table S7) and verified by sequencing (GATC Bio-tech).

All enzymes were expressed in *E. coli* BL21(DE3) strain in TB-medium. Expression was initiated with 0.1 mM IPTG, and after 16 h (24 h for psychrophilic enzymes) incubation at 25 °C (18 °C) cells were harvested (15 min at 7000 × g). For purification 1–4 g cells were resuspended in 5 volumes of 20 mM potassium phosphate (KP_i_)-buffer at pH 7, disrupted via sonification, and after centrifugation (15 min at 12000 × g) the supernatant was loaded on a NiNTA-column in a cyclic process (15 min). The column was connected to an ÄKTA purification system and after washing with KP_i_-buffer and 20 mM imidazole the enzyme was eluted with 250 mM imidazole. PD-10 desalting columns were used to rebuffer the solution to 20 mM KP_i_, and purity was verified with SDS-PAGE. Finally the protein concentration was determined by the Bradford assay^45^. Prior to crystallization screenings and to determine its hydrodynamic radii size exclusion chromatography was used. A SuperdexTM200 10/300 GL (GE Healthcare) column was equilibrated in 100 mM KP_i_, 150 mM NaCl (pH 7); 1–5 mg protein was loaded on the column, and the elution profile was recorded by means of absorbance at 280 nm. Five protein standards between 12.5 and 200 kDa were used for calibration (see Supplementary Fig. S5).

### Biochemical characterization

The activity for cleavage of the natural substrate 2-deoxy-D-ribose-5-phosphate (DRP) was measured for 1 min and (if not given explicitly) at 25 °C. In a retro-aldol reaction glyerinaldeyhde-3-phosphate is formed. This is reduced to glycerol-3-phosphate by the auxiliary-enzymes glycerol-3-phosphate dehydrogenase/triose phosphate isomerase (GDH/TPI) under NADH-consumption, which was detected with a photometer at 340 nm. The standard reaction mix (400 μl) contained 0.4 mM DRP, 0.15 mM NADH, 4 U GDH, 11 U TPI and 10 μl DERA solution.

Circular dichroism (CD) spectroscopy was used to determine the melting points of the proteins. The minimum of the far-UV spectrum at 222 nm was used to quantitate the folding of the proteins at different temperatures. A range from 25 °C to 100 °C was used with a heating rate of 1 °C/min. The molar ellipticity was plotted against temperature, resulting in a sigmoid curve, and the inflection point was used as the melting point.

### Determination of enantiomeric excess for a DERA-catalyzed aldol reaction between acetaldehyde and propanal

The aldol reaction was performed in analytical scale, followed by a derivatization with 2,4-DNPH. The products were extracted and analyzed by chiral HPLC. The DERA reaction was performed in 2 ml tubes. 28 μl of a stock solution containing 7 μl of propanal (0.10 mmol), 7 μl of acetaldehyde (0.12 mmol, 1.3 eq.) and 14 μl dimethyl sulfoxide (0.2 mmol, 2.0 eq.) were added to 0.5 ml of triethanolamine buffer (0.1 M, pH 7). After addition of 20 μl enzyme solution (cell free crude extract), the solutions were shaken at 25 °C and 200 rpm for 16 h. For derivatization a solution of 40 mg DNPH (0.2 mmol) and 7 μl conc. HCl (0.31 mmol) in 0.5 ml dimethyl sulfoxide was added to the DERA reaction. After stirring for 2 h at 50 °C, 300 rpm, the products were extracted with 600 μl of ethyl acetate. A short centrifugation led to good phase separation. 450 μl of the organic phase were transferred to another tube and dried over MgSO_4_ to avoid water contamination. After another short centrifugation step, 350 μl were transferred into new tubes. The organic phase was removed in a vacuum centrifuge. The residues were dissolved in 90:10 *n*-heptane/2-propanol-mixtures. To remove unsolved compound a short centrifugation was necessary. The clear yellow supernatant was then used to analyze the enantiomeric composition of the products by means of chiral HPLC: Chiralpak IA, 0.5 ml·min^−1^, 80:20 *n*-heptane:*i*PrOH, 345 nm: (*R*,*E*)-1-[2–(2,4-Dinitrophenyl)hydrazono]pentan-3-ol t_*R*_ = 70.9, (*S*,*E*)-1–[2-(2,4-Dinitrophenyl)hydrazono]pentan-3-ol t_*S*_ = 79.0.

### Synthesis of reference compounds

Details on the synthesis of the reference compounds as well as general information for chemical reactions are described in Supplementary Methods. A reaction schema is provided in Supplementary Fig. S6.

### Protein crystallization

Screening for crystallization conditions of psychrophilic DERAs was performed at 20 °C by sitting-drop vapor diffusion experiments, using a robotic system (Freedom EVO, Tecan). First crystals were generally observed after 1–3 days, with reservoir solutions containing 30% (w/v) PEG4000, 0.2 M lithium sulfate, 0.1 M Tris-HCl pH 8.5 (DERA_CP_, tetragonal form), 20% (w/v) PEG1000, 0.2 M calcium acetate, 0.1 M imidazole pH 8.0 (DERA_CP_, hexagonal form), or 30% (w/v) PEG6000, 1.0 M lithium chloride, 0.1 M sodium acetate (DERA_SH_), and protein concentrations of 10–15 mg/ml. In case of DERA_SH_, the initial condition was subjected to optimization, yielding well-diffracting samples with 27% (w/v) PEG6000, 1.0 M lithium chloride, 0.1 M sodium acetate, 0.1 M MES pH 6.0, and a 6.75 mg/ml protein solution. Prior to flash-cooling, crystals of DERA_CP_ were soaked in reservoir buffer containing up to 15% (v/v) glycerol; crystals of DERA_SH_ were processed in their mother liquor.

### Diffraction data collection and structure determination

X-ray diffraction datasets were recorded at 100 K, using beamlines ID23–1 and ID29 of the European Synchrotron Radiation Facility (ESRF; Grenoble, France) equipped with PILATUS 6 M detectors (Dectris). Data processing was performed with XDS and XSCALE^46^. Initial phases for the tetragonal crystal form of DERA_CP_ (space group P4_1_2_1_2) were determined by molecular replacement with MOLREP^47^, using chain A of DERA_EC_ (PDB ID1JCL) as a template. The asymmetric unit was found to contain two protein chains, corresponding to a Matthews coefficient of 2.69 Å^3^/Da and a solvent content of 54.3%. Following automated rebuilding in phenix.autobuild^48^, the model was further improved by alternating reciprocal space refinement in phenix.refine^49^ with rebuilding in Coot^50^. The final model served as template to determine the structures of DERA_SH_ (space group C2) and the hexagonal form of DERA_CP_ (space group P6_1_22) by molecular replacement. Both crystal forms contained four chains (two dimers) per asymmetric unit, with Matthews coefficients of 2.17 and 3.42 Å^3^/Da and solvent contents of 43.4 and 64.1%, respectively. For statistics of data collection and refinement, refer to Supplementary Table S1. Validation with MolProbity^51^ revealed good geometry, with all of the residues in the allowed regions of the Ramachandran plot.

### Generating structural ensembles by MD simulations

To generate a structural ensemble for subsequent CNA calculations, MD simulations were performed with Amber14 using the ff14SB force field^52^. DERA wt from *E. coli* (1JCL), *T. maritima* (3R12), and *P. aerophilum* (1VCV) were obtained from the Protein Data Bank. For each protein, three independent MD simulations (initiated by slightly different equilibration temperatures of T = 299.5 K, 300.0 K, and 300.5 K) of 100 ns length at 300 K (using a timestep of 2 fs) were performed. Except for the first 5 ns, conformations were extracted every 40 ps. This resulted in 1125 conformations for the first and 1250 for the last 50 ns. Preparation, minimization and equilibration of the system were done according to literature^22^. The analysis of the MD trajectories was carried out with cpptraj^53^ of AmberTools^23^. The RMSD of C_α_ atoms with respect to the crystal structure was computed as a measure of structural similarity, as was the radius of gyration of C_α_ atoms as a measure of structural compactness. Furthermore, the RMSIP as a measure of the amount of overlap of the conformational subspaces of two MD trajectories was calculated as described in literature^54^. The calculations were performed over the first 10 eigenvectors of a principal component analysis of the C_α_ atom covariance matrix of the atomic positional fluctuations^23^. Prior to computing the covariance matrix, the conformations were superimposed considering only those 80% of the residues with the lowest r.m.s. fluctuations to avoid introduction of spurious correlations^55^,^56^.

### Constraint network analysis (CNA)

Details on analyzing the thermostability of DERA with CNA are described in Supplementary Methods.

## Additional Information

**How to cite this article**: Dick, M. *et al*. Trading off stability against activity in extremophilic aldolases. *Sci. Rep.* **6**, 17908; doi: 10.1038/srep17908 (2016).

## Supplementary Material

Supplementary Information
