# Corrigendum: Trading off stability against activity in extremophilic aldolases

**DOI:** 10.1038/srep36431

**Published:** 2016-11-11

**Authors:** Markus Dick, Oliver H. Weiergräber, Thomas Classen, Carolin Bisterfeld, Julia Bramski, Holger Gohlke, Jörg Pietruszka

Scientific Reports 6: Article number: 1790810.1038/srep17908; published online: 01 19 2016; updated: 11 11 2016

The Supplementary Information file originally published with this Article omitted the PDB codes in Table S1. This error has now been corrected in the Supplementary Information file that accompanies the Article.

