# Supplementary material for: Trading off stability against activity in extremophilic aldolases
Source: Sci Rep. 2016 Jan 19;6:17908. doi: 10.1038/srep17908 (PMC4725968; doi:10.1038/srep17908)
Supplement: Supplementary Information [file srep17908-s1.pdf]

Supplementary Information

Trading off stability against activity in extremophilic aldolases

Markus Dick,[1] Oliver H. Weiergräber,[2] Thomas Classen,[3] Carolin Bisterfeld,[1] Julia Bramski,[1] Holger Gohlke[4,*] and Jörg Pietruszka[1,3,*]

[1]Institute of Bioorganic Chemistry, Heinrich-Heine-Universität Düsseldorf im Forschungs-zentrum Jülich, , and Bioeconomy Science Center (BioSC), Jülich, Germany

[2]Institute of Complex Systems ICS-6: Structural Biochemistry, Forschungszentrum Jülich GmbH, Jülich, Germany

[3]Institute of Bio- and Geosciences IBG-1: Biotechnology, Forschungszentrum Jülich GmbH, Jülich, Germany

[4]Institute of Pharmaceutical and Medicinal Chemistry, Heinrich-Heine-Universität Düsseldorf, Düsseldorf, Germany

[*]Correspondence to: Jörg Pietruszka, e-mail: j.pietruszka@fz-juelich.de; Holger Gohlke, e-mail: gohlke@uni-duesseldorf.de

Contents:

1. Supplementary Figures S1-S6
2. Supplementary Tables S1-S7
3. Supplementary Methods
4. Supplementary References

Supplementary Figures

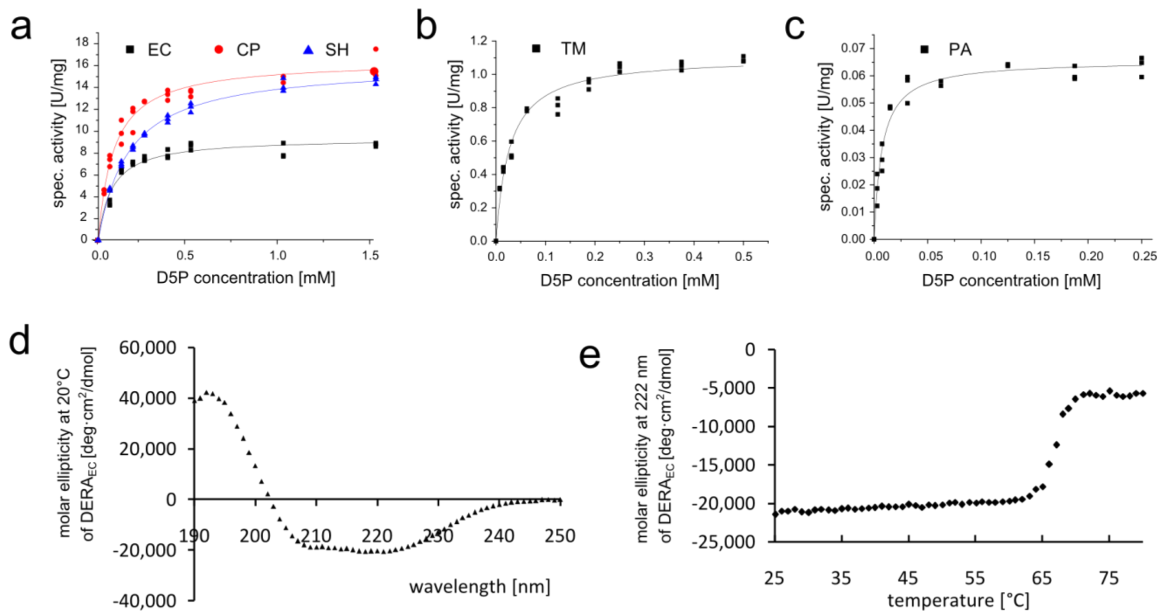

f

	30 °C	40 °C	50 °C	60 °C	70 °C	80 °C	90 °C	100 °C
CP	> 16 h	29 min	1 min	< 1 min	--	--	--	--
SH	> 16 h	6.6 h	1 min	< 1 min	--	--	--	--
EC	--	--	> 16 h	11 min	1 min	< 1 min	--	--
TM	--	--	--	--	--	> 16 h	13.6 h	22 min
PA	--	--	--	--	--	> 16 h	> 16 h	> 16 h

Supplementary Figure S1. Determination of Michaelis-Menten parameters and thermostability for all five DERA orthologs**.** (a)-(c) Michaelis-Menten kinetics: triplicate measurements were performed at 25 °C with different 2-deoxy-D-ribose-5-phosphate (D5P) concentrations. (d) Far-UV CD scan and (e) melting point determination for $DERA_{EC}$. Since the spectrum is dominated by α-helices, a minimum at 222 nm is observed. This was used to determine the melting point of the enzyme. (f) Activity half-life of DERA orthologs after thermal incubation. At specific time-points samples were taken for kinetic measurements at 25 °C. Then the half-life was calculated assuming a first order exponential decay function.

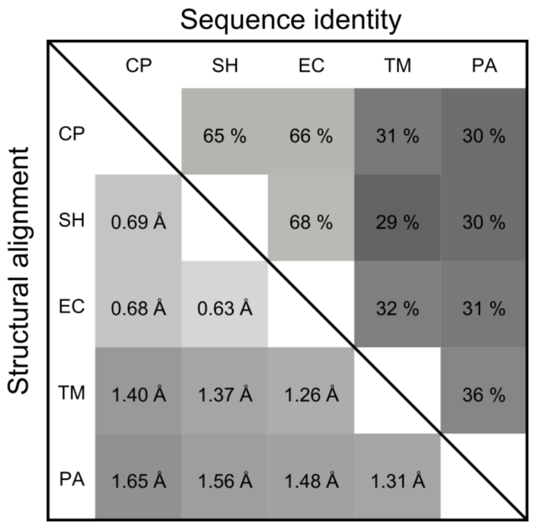

Supplementary Figure S2. Pairwise structural and sequence similarity of DERA orthologs, given as Cα-RMSD and percent sequence identity, respectively. Comparison is based on monomeric units (chain A) in all cases. Structures of *E. coli* (1JCL), *T. maritima* (3R12) and *P. aerophilum* (1VCV) were retrieved from the *Protein Data Bank*. Blosum62 matrix was used for sequence alignments.

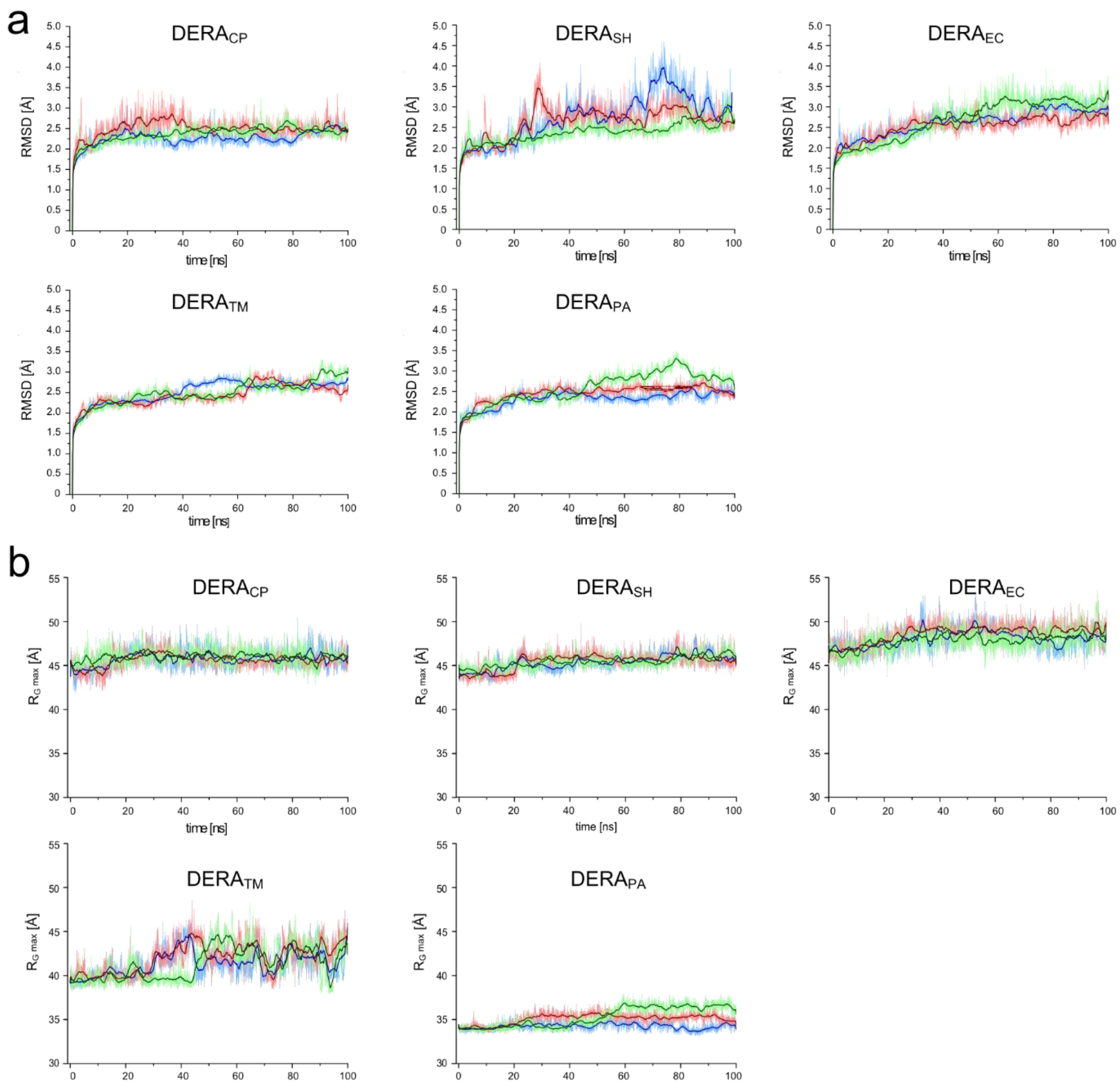

Supplementary Figure S3. (a) Root mean-square deviations with respect to the starting structure and (b) radius of gyration (R_g) for C_α atoms computed from three independent MD simulations of DERAs from *C. psychrerythraea*, *S. halifaxensis*, *E. coli*, *T. maritima*, and *P. aerophilum*. PDB codes are given in Table S2. In each case, 5000 conformations over 100 ns of simulation time were considered. The darker lines represent the moving average of 100 conformations. For the radius of gyration average values over all conformations (± standard deviation) were determined to be 45.8±1.0 Å ($DERA_{CP}$), 45.4±0.9 Å ($DERA_{SH}$), 48.1±1.2 Å ($DERA_{EC}$), 41.5±1.9 Å ($DERA_{TM}$) and 34.9±0.9 Å ($DERA_{PA}$).

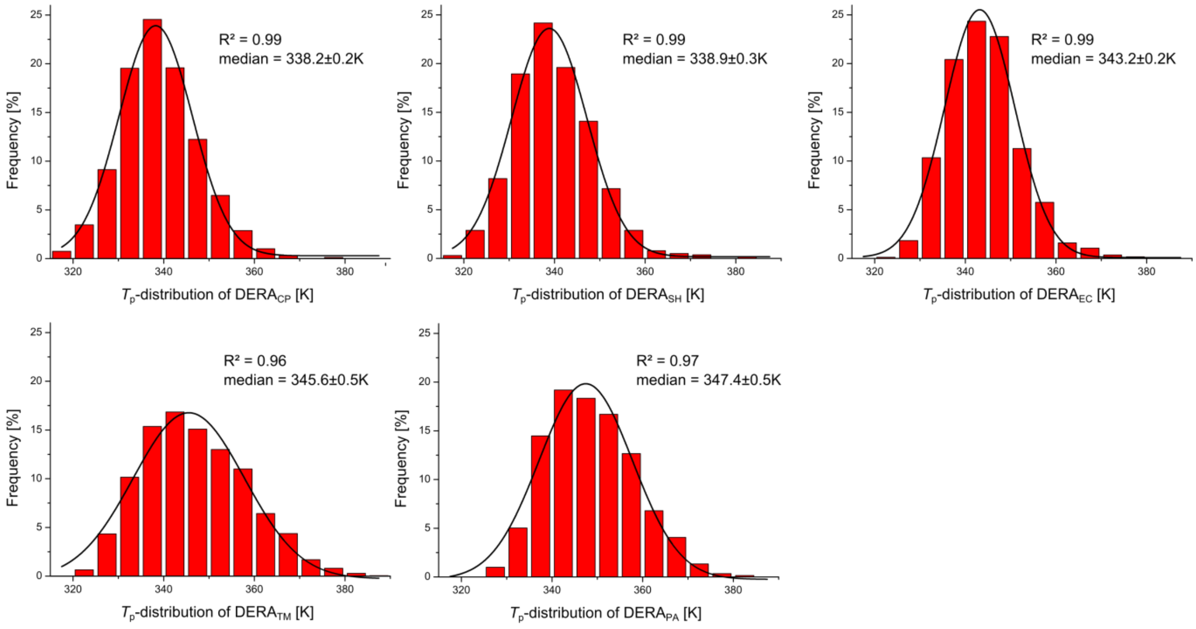

Supplementary Figure S4. Distribution of transition points (T_p) derived from constraint network analysis, based on ensembles of 3 x 1125 conformations generated by MD simulations. The black curves represent the fitted Gaussians; correlation coefficients (R^2) and medians with standard errors are indicated.

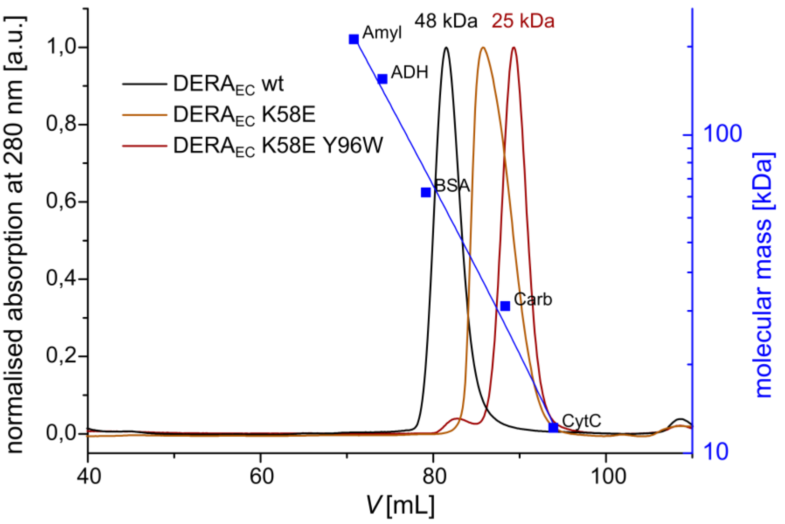

Supplementary Figure S5. Size exclusion chromatogram of different $DERA_{EC}$ variants. A logarithmic calibration curve (blue) with protein standards β-amylase (Amyl, 200 kDa), alcohol dehydrogenase (ADH, 150 kDa), bovine serum albumin (BSA, 66 kDa), carbonic anhydrase (Carb, 29 kDa) and cytochrome C (CytC, 12.4 kDa) was used to determine the apparent molecular mass. The *in silico* mass of wt $DERA_{EC}$ (single chain) equals 28.6 kDa.

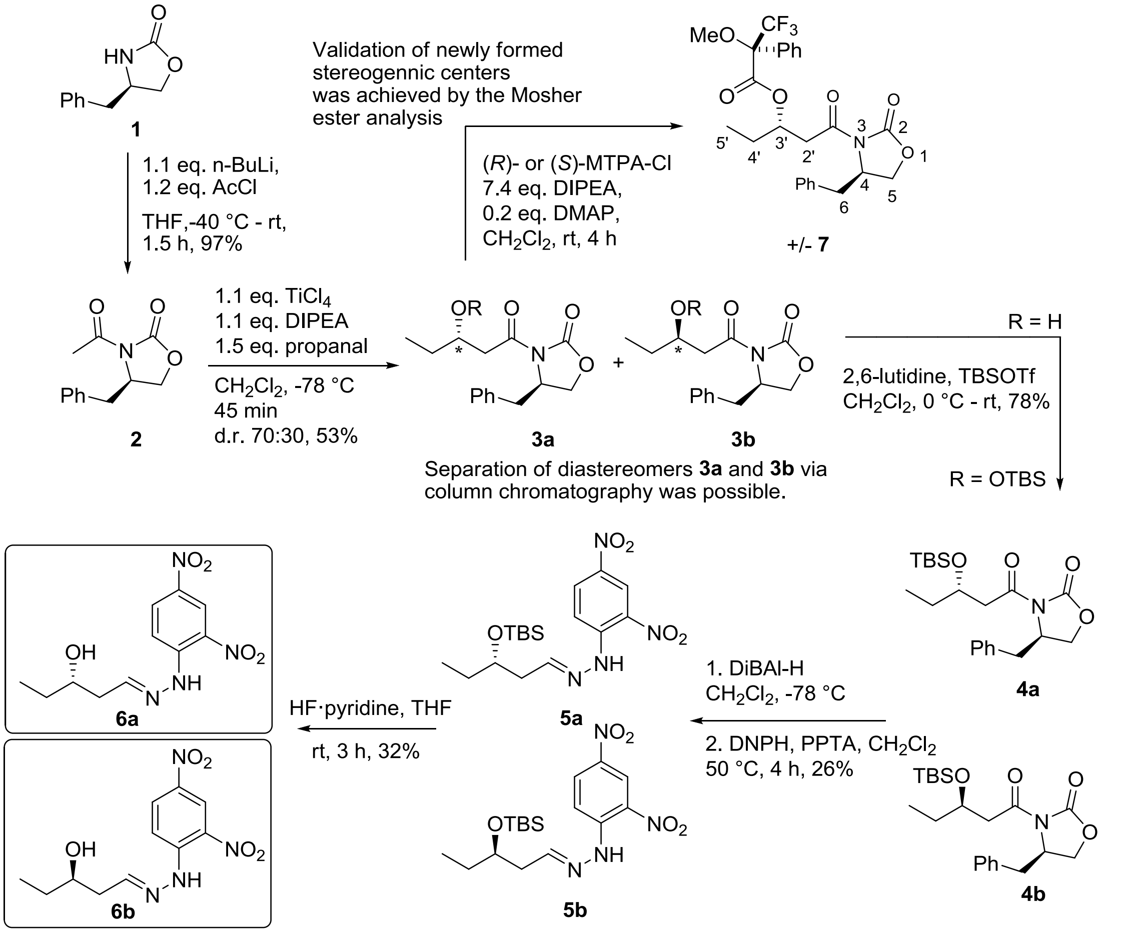

Supplementary Figure S6. Synthesis of the reference compounds **6a** and **6b** for HPLC measurements.

Supplementary Tables

Table S1. Data collection and refinement statistics

	$DERA_{CP}$ (tetragonal form)	$DERA_{CP}$ (hexagonal form)	$DERA_{SH}$
**Data collection**			
Space group	$P4_12_12$	$P6_122$	C2
Cell dimensions			
a, *b*, *c* (Å)	103.76, 103.76, 112.98	115.84, 115.84, 400.10	154.03, 52.51, 143.21
α, β, γ (°)	90.00, 90.00, 90.00	90.00, 90.00, 120.00	90.00, 122.34, 90.00
Resolution (Å)	47.15-2.11 (2.16-2.11) *	49.77-2.10 (2.15-2.10)	48.70-1.76 (1.81-1.76)
$CC_{1/2}$ (%)	99.8 (56.4)	99.9 (87.5)	99.8 (70.5)
R_{meas} (%)	12.9 (98.6)	9.1 (118.8)	7.8 (80.1)
$I/\sigma I$	12.2 (2.0)	21.4 (2.6)	12.3 (2.0)
Completeness (%)	82.3 (82.2)	99.6 (97.8)	99.4 (99.6)
Redundancy	6.9 (6.5)	13.4 (12.8)	4.1 (4.2)
**Refinement**			
Resolution (Å)	47.15-2.11	49.77-2.10	48.70-1.76
No. reflections	29929	93105	95902
R_{work} / R_{free} (%)	16.1 / 20.1	17.3 / 19.9	16.8 / 19.8
No. atoms			
Protein	3611	7389	7361
Ligands/ions	26	48	6
Water	303	438	922
B-factors (Å^2)			
Protein	33.4	50.4	39.2
Ligand/ion	45.0	69.3	34.0
Water	42.0	48.5	42.5
R.m.s. deviations			
Bond lengths (Å)	0.005	0.006	0.006
Bond angles (°)	0.813	0.903	0.927

*Values in parentheses are for highest-resolution shell.

Table S2. Overview of PDB codes used for the calculations

organism	PDB code	amino acids (both chains)
C. psychrerythraea	5C2X	492
S. halifaxensis	5C6M	492
E. coli	1JCL	503
T. maritima	3R12	510
P. aerophilum	1VCV	452

Table S3. Number of non-covalent interactions in DERA variants and correlation to optimal growth temperatures of their hosts. The FIRST software was used for these calculations with either the crystal structures (dimers) or an ensemble of conformations derived from 3 x 50 ns of MD simulation as input. Input files for the structures are listed in Table S2.

			DERA$_{CP}$	DERA$_{SH}$	DERA$_{EC}$	DERA$_{TM}$	DERA$_{PA}$		
optimal growth temperature of organism			8 °C	10 °C	37 °C	80 °C	100 °C	slope	R^2
crystal structures	hydrogen bonds and salt bridges	<-0.1 kcal/mol	511	524	562	567	497	0.02	0.00
		<-1 kcal/mol	406	406	442	447	381	-0.05	0.01
		<-2 kcal/mol	339	361	386	392	322	-0.06	0.01
		<-3 kcal/mol	287	280	316	343	254	0.01	0.00
		<-4 kcal/mol	207	198	226	261	175	0.03	0.00
		<-5 kcal/mol	122	114	143	170	108	0.13	0.05
		<-6 kcal/mol	63	62	79	92	66	0.14	0.22
	hydrophobic interactions		201	225	226	297	294	1.01	0.91
ensemble from MD simulations	hydrogen bonds and salt bridges	<-0.1 kcal/mol	556±11	552±12	586±13	612±11	531±10	0.06	0.01
		<-1 kcal/mol	406±10	402±10	431±11	453±10	396±9	0.13	0.06
		<-2 kcal/mol	321±10	316±11	342±10	361±11	316±10	0.15	0.10
		<-3 kcal/mol	248±10	243±10	267±10	282±10	249±10	0.18	0.19
		<-4 kcal/mol	181±9	177±10	199±9	212±9	189±9	0.21	0.37
		<-5 kcal/mol	121±8	120±8	139±8	150±9	137±8	0.25	0.62
		<-6 kcal/mol	73±6	75±7	90±7	100±7	96±7	0.28	0.84
	hydrophobic interactions		192±14	190±13	213±15	247±17	184±14	0.18	0.08

Table S4. Root mean-square inner product (RMSIP) over the first ten principal components for respective pairs of independent MD simulations of one DERA ortholog.*

Source	DERA$_{CP}$			DERA$_{SH}$			DERA$_{EC}$			DERA$_{TM}$			DERA$_{PA}$		
MD	1	2	3	1	2	3	1	2	3	1	2	3	1	2	3
1	1.00	0.75	0.77	1.00	0.79	0.75	1.00	0.69	0.71	1.00	0.67	0.63	1.00	0.75	0.68
2	0.75	1.00	0.77	0.79	1.00	0.78	0.69	1.00	0.73	0.67	1.00	0.68	0.75	1.00	0.66
3	0.77	0.77	1.00	0.75	0.78	1.00	0.71	0.73	1.00	0.63	0.68	1.00	0.68	0.66	1.00

*RMSIP values range from 0 to 1, with 1 indicating completely overlapping conformational subspaces and 0 completely independent conformational subspaces; the submatrices are symmetric with comparisons of an MD trajectory to itself on a diagonal.

Table S5. Average phase transition points T_p with standard errors of the mean from CNA analyses on MD simulation-derived ensembles. The standard error of the mean for averaging over all three MD simulations was computed from the single standard errors of the mean considering laws of error propagation. Input files for the structures are listed in Table S2.

Source	Phase transition points T_p with standard error [°C]							
	5-50 ns				50-100 ns			
	MD1	MD2	MD3	Mean	MD1	MD2	MD3	Mean
DERA$_{CP}$	65.3±0.30	66.5±0.29	66.2±0.29	66.0±0.51	65.3±0.29	66.9±0.31	63.9±0.26	65.4±0.50
DERA$_{SH}$	68.7±0.33	66.7±0.31	65.4±0.29	66.9±0.54	65.3±0.32	64.4±0.28	66.2±0.32	65.3±0.53
DERA$_{EC}$	70.0±0.27	71.2±0.28	71.8±0.28	71.0±0.48	68.5±0.26	68.7±0.26	69.2±0.24	68.8±0.44
DERA$_{TM}$	73.8±0.38	72.9±0.44	74.9±0.36	73.9±0.68	72.6±0.37	73.0±0.37	75.9±0.32	73.8±0.61
DERA$_{PA}$	76.3±0.31	73.1±0.31	77.0±0.32	75.5±0.54	75.4±0.30	75.2±0.27	74.3±0.26	75.0±0.48

Table S6. Specific activity and activity based stability of dimeric vs. monomeric DERA variants

	spec. activity (U/mg)		incubation temperature	$T_{1/2}$	
	dimer	monomer		dimer	monomer
CP	10.33±0.94	9.76±0.63	45°C	6.0 min	3.3 min
SH	11.51±0.69	11.76±0.47	45°C	9.6 min	3.1 min
EC	7.5±0.32	5.87±0.52	60°C	10.7 min	4.9 min
TM	1.09±0.04	1.08±0.02	90°C	>16 h	<1 min
			80°C	>16 h	4.1 min
			70°C	>16 h	28.7 min

Table S7. Primer sequences used for site-directed mutagenesis.
Mutation sites are labeled in red.

**name**	**5'-3'-sequence**
isolation of deoC$_{SH}$ from genomic DNA of *S. halifaxensis*	
deoC$_{SH}$_fw	CATATGAGCGACTTAAAAAAAGCAGC
deoC$_{SH}$_rv	GACTCGAGGTAACCTTGTGCACCTTG
Mutants to design monomeric DERA-orthologs	
deoC$_{EC}$_K58E_fw	[PHOS]GAAACTCTGAAAGAGCAGGGCACCCCGG
deoC$_{EC}$_K58E_rv	[PHOS]GCGAGCAATCGGGATAAAGCGAGGAT
deoC$_{EC}$_Y96W_fw	[PHOS]TGGGGTGCTGATGAAGTTGACGTTGTGTTCC
deoC$_{EC}$_Y96W_rv	[PHOS]GGCGATTGCCGCACGGGTTTCTGCC
deoC$_{CP}$_Y96W_fw	GGGGCGCTGATGAAGTCGATTTAGTCT
deoC$_{CP}$_Y96W_rv	AAGCGACAGCAGCACGTGTTTCTG
deoC$_{SH}$_Y96W_fw	CAGCAGTTGCATGGGGCGCCGACGAAG
deoC$_{SH}$_Y96W_rv	CTTCGTCGGCGCCCCATGCAACTGCTG
deoC$_{TM}$_L93E_fw	CGTTGTTGGTTTTCCAGAGGGAGCGAACGAAAC
deoC$_{TM}$_L93E_rev	GTTTCGTTCGCTCCCTCTGGAAAACCAACAACG
deoC$_{TM}$_E104W_fw	GGACGAAAGCCCATTGGGCGATTTTCGCTG
deoC$_{TM}$_E104W_rev	CAGCGAAAATCGCCCAATGGGCTTTCGTCC
deoC$_{TM}$_L99E_fw	[PHOS]GAGACGAAAGCCCATTGGGCGATTTTC
deoC$_{TM}$_L99E-R93E_rv	[PHOS]AGTTTCGTTCGCTCCCTCTGGAAAACC
deoC$_{TM}$_L99E_rv	[PHOS]AGTTTCGTTCGCTCCCAGTGGAAAACC
deoC$_{PA}$_F61W_fw	GTGGCGGACTTCCCCTGGGGGGCCTTGCCAAC
deoC$_{PA}$_F61W_rev	GTTGGCAAGGCCCCCCAGGGGAAGTCCGCCAC
deoC$_{PA}$_Y156I_fw	TTCGCCGAAGAGGCCATCGCCGCCAGACAGGGA
deoC$_{PA}$_Y156I_rv	TCCCTGTCTGGCGGCGATGGCCTCTTCGGCGAA
deoC$_{PA}$_R75L_fw	[PHOS]CTGCTTGCTGAAGTGGCAGATGAGATAGAC
deoC$_{PA}$_R75L_rv	[PHOS]AGAAACCAAGGCAATTCTGCTGGCCGTTG
deoC$_{PA}$_I39A_fw	ATTGCGTAAATCCCGCATACGCCCCTGTTGT
deoC$_{PA}$_I39A_rv	ACAACAGGGGCGTATGCGGGATTTACGCAAT
Introduction of intermolecular disulfide bridge	
deoC$_{EC}$_A95C_fw	[PHOS]GCTACGGTGCTGATGAAGTTGACGTTGT
deoC$_{EC}$_A95C_rv	[PHOS]AGATTGCCGCACGGGTTTCTGCCA
deoC$_{CP}$_A95C_fw	ACGTGCTGCTGTCTGTTATGGCGCTGATG
deoC$_{CP}$_A95C_rv	CATCAGCGCCATAACAGACAGCAGCACGT
deoC$_{SH}$_A95C-fw	TCGCGCAGCAGTTTGTTACGGCGCCGACGA
deoC$_{SH}$_A95C-rv	TCGTCGGCGCCGTAACAAACTGCTGCGCGA

Supplementary Methods

Constraint network analysis (CNA)

In the CNA approach, a protein is modeled as a constraint network, where bodies (representing atoms) are connected by sets of bars (constraints, representing covalent and noncovalent interactions)[1]. A rigidity analysis performed on the network[2,3] results in a decomposition into rigid parts and flexible links in between. By analyzing a series of "perturbed" networks in which noncovalent interactions are included in a temperature-dependent manner[4-6] the loss of rigidity of a protein is simulated, which can be related to thermal unfolding[4,6,7]. Here, hydrogen bonds with an energy E_{HB} (computed according to a modified version[6] of the potential by Mayo and coworkers[8].) larger (i.e., less negative) than a certain cutoff E_{cut} were removed from the network in a stepwise manner. E_{cut} was varied between −0.1 kcal mol^{-1} and −6.0 kcal mol^{-1} with a step size of 0.1 kcal mol^{-1}. According to the linear relationship between E_{cut} and the temperature T introduced previously[4,7], the range of E_{cut} used in this study is equivalent to increasing the temperature of the system from 302 K to 420 K with a step size of 2 K. Results of these analyses can be linked to biologically relevant characteristics of a biomolecular structure by a set of global and local indices[9]. In particular, a phase transition point T_p can be identified during the thermal unfolding simulation at which a largely rigid network becomes almost flexible; this phase transition point has been related to the thermodynamic thermostability of a protein[4,5,7]. T_p was identified by a modified cluster configuration entropy H_{type2}[4,9]. H_{type2} monitors the degree of disorder in the realization of a given network state during the unfolding simulation. The H_{type2} *versus* T curve obtained from a thermal unfolding simulation was fitted with a double sigmoid as done previously [5], and the temperature T_p was identified as the inflection point of the sigmoid with the larger difference in the asymptote values.

For characterizing the local rigidity and to understand the influence of mutations on DERA properties at a microscopic level, the stability map rc_{ij} was used[7]. A stability map is derived by identifying "rigid contacts" between two residues i and j that are represented by their C_α atoms. A rigid contact exists if the two residues belong to the same rigid cluster. During a thermal unfolding simulation, stability maps are then constructed: for each residue pair, E_{cut} (or, equivalently, a temperature derived from the relationship $T = f(E_{cut})$ described in literature[4,7]) is identified at which a rigid contact between these residues is lost. When filtered by considering only rigid contacts between residues that are at most 5 Å apart from each other (measured as the distance between the closest atom pair of the two residues), a neighbor stability map results[10]. This map helps focusing on short-range residue contacts. For improving the robustness of the rigidity analyses, they are performed on structural ensembles generated by MD simulations. Results are averaged over the ensembles, and the uncertainty is computed by the standard error of the mean.

Synthesis of reference compounds for the DERA-catalyzed aldol reaction

General information for chemical reactions

All reactions were carried out using standard Schlenk techniques under dry nitrogen with magnetic stirring. Glassware was oven dried at 120 °C overnight. Solvents were dried and purified by conventional methods prior to use. All reagents were used as purchased from commercial suppliers unless otherwise specified. Common solvents for chromatography [*n*-pentane, petroleum ether (bp 40–60 °C), ethyl acetate] were distilled prior to use. Flash column chromatography was performed on silica gel 60, 0.040–0.063 mm (230–400 mesh). Thin layer chromatography (TLC; monitoring the

course of the reaction) was performed on pre-coated plastic sheets with detection by UV (254 nm) and/or by coloration with cerium molybdenum solution [phosphomolybdic acid (25 g), $Ce(SO_4)_2 \cdot H_2O$ (10 g), conc. H_2SO_4 (60 ml), H_2O (940 ml)]. ^1H and ^{13}C NMR spectra were recorded at room temperature in $CDCl_3$ on a spectrometer at 600 and 151 MHz respectively, relative to internal standard tetramethylsilane [TMS; ^1H: δ(TMS) = 0.00] or relative to the resonance of the solvent [^{13}C: δ($CDCl_3$) = 77.0]. Higher order δ and *J* values are not corrected. ^{13}C signals were assigned by means of C, H, COSY and HSQC spectroscopy. Optical rotations were measured using a quartz cell with 1 ml capacity and a 10 cm path length. IR data were measured on a PerkinElmer SpectrumOne instrument as thin film. Absorbance frequencies are reported in cm^{-1}. Melting points are uncorrected. Elemental analyses and HRMS measurements were performed by the analytical service of the Forschungszentrum Jülich.
HPLC Measurements were performed on a Dionex instrument consisting of a LPG-3400A pump, a WPS-3000TSL autosampler, a DAD-3000 UV-detector and a TCC-3000SD oven. The instrument was fitted with a Daicel Chiralpak IA (250 mm x 4.6 mm), adjusted to 25 °C. As solvent an 80:20 *n*-heptane/2-propanol-mixture was used applying a flow rate of 0.5 ml min^{-1}. Compounds were solved in 90:10 *n*-heptane/2-propanol-mixtures and measured at 345 nm.
DNPH refers to 2,4-dinitrophenylhydrazin, MTPA-Cl refers to α-methoxy-α-trifluoro-methylphenylacetic acid chloride, TBSOTf refers to *tert*-butyldimethylsilyl triflate, PTSA*H_2O refers to *p*-toluenesulfonic acid monohydrate.

Numbering of the compounds corresponds to Fig. S5

(4*R*)-3-Acetyl-4-benzyloxazolidin-2-one (**2**)

(*R*)-4-Benzyl-2-oxazolidinon (**1**) (1.25 g, 7.05 mmol) were solved in 15 ml tetrahydrofuran and cooled to -40 °C. Afterwards 3.1 ml *n*-butyllithium (7.76 mmol, 2.5 mol/L in *n*-hexane, 1.1 eq.) were added dropwise, leading to a white precipitate. After 15 min freshly distilled acetylchloride (0.61 ml, 8.47 mmol, 1.2 eq.) was added and the reaction was stirred for 60 min at -40 °C. The reaction was quenched by addition of 5 ml 2 M HCl and 5 ml H_2O and warmed up to room temperature (40 min). The reaction mixture was extracted with diethylether (3x), the combined organic phases were washed with brine and dried over $MgSO_4$. Evaporation of the solvent resulted in 1.54 g (7.02 mmol, 99.5%) of product **2** as a white solid.

TLC: R_f = 0.18 (petroleum ether:ethyl acetate 80:20).

GC-MS (EI, positive-Ion, 70 eV): t_R = 10.95 min, m/z (%) 219 (40) [M^+], 128 (60) [$(M-PhCH_2)^+$], 91 (30) [Ph^+], 86 (100) [$(M-C_9H_{10}O)^+$], 77 (10), 65 (30), 51 (10).

Elemental analysis (%) calculated for $C_{12}H_{13}NO_3$: C 65.74, H 5.98, N 6.39, found: C 66.24 ± 0.09, H 5.98 ± 0.05, N 6.61 ± 0.14.

Melting point: 108 °C.

IR (Film): $\tilde{\nu}$[cm^{-1}] = 3068, 3028, 2991, 2955, 1773, 1694, 1492, 1455, 1388, 1370, 1350, 1284, 1215, 1135, 1048, 972, 739, 699.

$[\alpha]_D^{20}$= -68.0 (c = 1.2, $CHCl_3$); Lit.: -100.3 (concentration and solvent are not specified)[11]

^{1}H NMR (600 MHz, $CDCl_3$): δ (ppm) = 2.56 (s, 3 H, 2'-H), 2.78 (dd, $^2J_{6a,6b}$ = 13.43 Hz, $^3J_{6a,4}$ = 9.6 Hz, 1 H, 6-H_a), 3.31 (dd, $^2J_{6b,6a}$ = 13.4 Hz, $^3J_{6b,4}$ = 3.4 Hz, 1 H, 6-H_b), 4.17 (dd, $^2J_{5a,5b}$ = 9.1 Hz, $^3J_{5a,4}$ = 3.2 Hz, 1 H, 5-H_a), 4.20 (dd, $^2J_{5b,5a}$ = 9.1 Hz, $^3J_{5b,4}$ = 7.7 Hz, 1 H, 5-H_b), 4.67 (dddd, $^3J_{4,6a}$ = 9.6 Hz, $^3J_{4,5b}$ = 7.7 Hz, $^3J_{4,5a}$ = 3.2 Hz, $^3J_{4,6b}$ = 3.4 Hz, 1 H, 4-H), 7.21-7.35 (m, 5 H, arom-H).

^{13}C NMR (151 MHz, $CDCl_3$): δ (ppm) = 23.8 (C-2'), 37.8 (C-6), 55.0 (C-4), 66.1 (C-5), 127.4/129.0/129.4 (Ph), 135.2 (C-7) 153.7(C=O), 170.3(C=O).

The analytical data are conform with the literature[11].

(4*R*,3'*S*)-4-Benzyl-3-hydroxypentanoyl-oxazolidin-2-one (**3a**) and (4*R*,3'*R*)-4-benzyl-3-hydroxypentanoyloxazolidin-2-one (**3b**)

1.00 g of compound **2** (4.56 mmol) were solved in 40 ml CH_2Cl_2 and cooled to -78 °C. Addition of 0.6 ml titan tetrachloride (5.47 mmol, 1.2 eq.) led to an orange color of the solution. After 5 min *N*,*N*-diisopropylethylamine (DIPEA, 1.01 ml, 5.93 mmol, 1.3 eq.) was slowly added. The color changed to black. After another 35 min, 0.5 ml (6.89 mmol, 1.51 eq.) propanal were added. The reaction was stirred for 3 h, quenched with saturated NH_4Cl_2 solution and diluted with dichloromethane. After extraction with dichloromethane (3x) the combined organic layers were washed once with brine and dried over $MgSO_4$. The diastereomeric ratio was 2:1. After evaporation of the solvent, the crude product was purified by column chromatography. 728 mg (2.63 mmol, 58%) of the main diastereomer **3a** and 215 mg (0.78 mmol, 17%) of the minor diastereomer **3b** (total yield 75%) could be isolated.

Main diastereomer (**3a**):

TLC: R_f = 0.39 (petroleum ether:ethyl acetate 60:40).

GC-MS (EI, positive-Ion, 70 eV): t_R = 12.34 min, m/z (%) 277 (15) [M^+], 248 (30) [$(M-C_2H_5)^+$].

Elemental analysis (%) calculated for $C_{15}H_{19}NO_4$: C 64.97, H 6.91, N 5.05, found: C 64.83 ±0.42, H 6.84 ± 0.09, N 5.16 ± 0.05.

Melting point: 44.6 °C.

IR (Film): $\tilde{\nu}$[cm^{-1}] = 3544, 2920, 2969, 1783, 1699, 1675, 1498, 1480, 1455, 1379, 1353, 1295, 1200, 1112, 1047, 1030, 978, 873, 760, 706.

$[\alpha]_D^{20}$= -24.0 (c = 0.9, $CHCl_3$).

^{1}H NMR (600 MHz, $CDCl_3$): δ (ppm) = 1.00 (t, $^3J_{5',4'}$ = 7.4 Hz, 3 H, 5'-H), 1.53-1.65 (m, 2 H, 4'-H), 2.81 (dd, $^2J_{6a,6b}$ = 13.5 Hz, $^3J_{6a,4}$ = 9.4 Hz, 1 H, 6-H_a), 2.97 (d, $^3J_{OH,3'}$ = 4.3 Hz, 1 H, OH), 3.05 (dd, $^2J_{2'a,2'b}$ = 17.4 Hz, $^3J_{2'a,3'}$ = 9.3 Hz, 1 H, 2'-H_a), 3.13 (dd, $^2J_{2'b,2'a}$ = 17.4 Hz, $^3J_{2'b,3'}$ = 2.8 Hz, 1 H, 2'-H_b), 3.29 (dd, $^2J_{6b,6a}$ = 13.5 Hz, $^3J_{6b,4}$ = 3.5 Hz, 1 H, 6-H_b), 4.04 (ddddd, $^3J_{3',2'a}$ = 9.3 Hz, $^3J_{3',4'}$ = 7.4 Hz, $^3J_{3',OH}$ = 4.3 Hz, $^3J_{3',4'}$ = 4.3 Hz, $^3J_{3',2'b}$ = 2.8 Hz, 1 H, 3'-H), 4.19 (dd, $^2J_{5a,5b}$ = 9.1 Hz, $^3J_{5a,4}$ = 3.0 Hz, 1 H, 5-H_a), 4.23 (dd, $^2J_{5b,5a}$ = 9.1 Hz, $^3J_{5b,4}$ = 7.6 Hz, 1 H, 5-H_b), 4.71 (dddd, $^3J_{4,6a}$ = 9.4 Hz, $^3J_{4,5b}$ = 7.6 Hz, $^3J_{4,6b}$ = 3.5 Hz, $^3J_{4,5a}$ = 3.0 Hz, 1 H, 4-H), 7.19-7.36 (m, 5 H, arom-H).

^{13}C NMR (151 MHz, $CDCl_3$): δ (ppm) = 9.9 (C-5'), 29.5 (C-4'), 37.8 (C-6), 42.3(C-2'), 55.1(C-4), 66.3 (C-5), 69.4(C-3'), 127.4/129.0/129.4 (Ph), 135.3 (C-7), 153.5(C=O), 173.0(C=O).

Minor diastereomer **(3b)**:

TLC: R_f = 0.30 (petroleum ether:ethyl acetate 60:40).

GC-MS (EI, positive-Ion, 70 eV): t_R = 10.93 min, m/z (%) 219 (50) [$(M-C_3H_7O)^+$], 91 (100) [Ph^+].

Elemental analysis (%) calculated for $C_{15}H_{19}NO_4$: C 64.97, H 6.91, N 5.05, found: C 65.14 ± 0.12, H 6.97 ± 0.02, N 4.94 ± 0.05.

Melting point: 83.1 °C.

IR (Film): $\tilde{\nu}$[cm^{-1}] = 3431, 2997, 2959, 1761, 1695, 1496, 1457, 1393, 1354, 1303, 1224, 1186, 1136, 1107, 1008, 984, 910, 756, 744, 716, 697.

$[\alpha]_D^{20}$ = -89.3 (c = 1.0, $CHCl_3$).

^{1}H NMR (600 MHz, $CDCl_3$): δ (ppm) = 1.01 (t, $^3J_{5',4'}$ = 7.4 Hz, 3 H, 5'-H), 1.53-1.66 (m, 2 H, 4'-H), 2.79 (dd, $^2J_{6a,6b}$ = 13.4 Hz, $^3J_{6a,4}$ = 9.6 Hz, 1 H, 6-H_a), 2.85 (d, $^3J_{OH,3'}$ = 4.2 Hz, 1 H, OH), 2.98 (dd, $^2J_{2'a,2'b}$ = 17.4 Hz, $^3J_{2'a,3'}$ = 9.5 Hz, 1 H, 2'-H_a), 3.19 (dd, $^2J_{2'b,2'a}$ = 17.4 Hz, $^3J_{2'b,3'}$ = 2.5 Hz, 1 H, 2'-H_b), 3.31 (dd, $^2J_{6b,6a}$ = 13.4 Hz, $^3J_{6b,4}$ = 3.4 Hz, 1 H, 6-H_b), 4.07 (m, 1 H, 3'-H), 4.19 (dd, $^2J_{5a,5b}$ = 9.1 Hz, $^3J_{5a,4}$ = 2.8 Hz, 1 H, 5-H_a), 4.2 (dd, $^2J_{5b,5a}$ = 9.1 Hz, $^3J_{5b,4}$ = 8.0 Hz, 1 H, 5-H_b), 4.7 (dddd, $^3J_{4,6a}$ = 9.6 Hz, $^3J_{4,5b}$ = 8.0 Hz, $^3J_{4,6b}$ = 3.4 Hz, $^3J_{4,5a}$ = 2.8 Hz, 1 H, 4-H), 7.20-7.36 (m, 5 H, arom-H).

^{13}C NMR (151 MHz, $CDCl_3$): δ (ppm) = 9.9 (C-5'), 29.5(C-4'), 38.0(C-6), 42.3(C-2'), 55.1 (C-4), 66.4(C-5), 69.1 (C-3'), 127.4/129.1/129.4 (Ph), 135.3 (C-7), 153.5(C=O), 173.0 (C=O).

Determination of the absolute configuration of (4*R,3'S*)-4-benzyl-3-hydroxypentanoyl-oxazolidin-2-one (**3a**) by 'Mosher ester analyses'[12]

20 mg of compound **3a** (0.07 mmol) and 1.8 mg 4-dimethylaminopyridine (0.01 mmol, 0.2 eq., 99%) were solved in 1.7 ml CH_2Cl_2 and stirred at room temperature. *N,N*-diisopropylethylamine (91 µl, 0.53 mmol, 7.4 eq.) and (*R*)- or (*S*)-MTPA-Cl (16 µl, 0.09 mmol, 1.2 eq.) were added. The reaction was stirred for 2 h and then diluted with ethyl acetate. The reaction was washed with 1 M H_3PO_4-solution (3x), saturated $NaHCO_3$-solution (2x) and saturated NaCl-solution (1x), dried over $MgSO_4$ and the solvent was evaporated. The crude product was measured by NMR.

(*S*)-7-MTPA ester

^{1}H NMR (600 MHz, $CDCl_3$): δ (ppm) = 0.86 (t, $^3J_{5',4'}$ = 7.4 Hz, 3 H, 5'-H), 1.74 (m, 2 H, 4'-H), 2.73 (dd, $^2J_{6a,6b}$ = 13.4 Hz, $^3J_{6a,4}$ = 9.6 Hz, 1 H, 6-H_a), 3.24 (dd, $^2J_{6b,6a}$ = 13.4 Hz, $^3J_{6b,4}$ = 3.5 Hz, 1 H, 6-H_b), 3.27 (dd, $^2J_{2a',2b'}$ = 17.0 Hz, $^3J_{2a',3'}$ = 8.8 Hz, 1 H, 2'-H_a), 3.31 (dd ($^2J_{2b',2a'}$ = 17.0 Hz, $^3J_{2b',3'}$ = 3.6 Hz, 1 H, 2'-H_b), 3.58 (s, 3 H, OMe), 4.17 (dd, $^2J_{5a,5b}$ = 9.0 Hz, $^3J_{5a,4}$ = 2.8 Hz, 1 H, 5-H_a), 4.24 (dd, $^2J_{5b,5a}$= 9.0 Hz, $^3J_{5b,4}$ = 9.0 Hz, 1 H, 5-H_b), 4.63 (dddd, $^3J_{4,6a}$ = 9.6 Hz, $^3J_{4,5b}$ = 9.0 Hz, $^3J_{4,6b}$ = 3.5 Hz, $^3J_{4,5a}$ = 2.8 Hz, 1 H, 4-H), 5.53 (dddd, $^3J_{3',2a'}$ = 8.8 Hz, $^3J_{3',4a'}$ = 5.95 Hz, $^3J_{3',4b'}$ = 5.9 Hz, $^3J_{3',2b'}$ = 3.6 Hz, 1 H, 3'-H), 7.14-7.60 (m, 10 H, arom-H).

(*R*)-7-MTPA ester

^{1}H NMR (600 MHz, $CDCl_3$): δ (ppm) = 1.00 (t, $^3J_{5',4'}$ = 7.4 Hz, 3 H, 5'-H), 1.82 (m, 2 H, 4'-H), 2.60 (dd, $^2J_{6a,6b}$ = 13.5 Hz, $^3J_{6a,4}$ = 9.6 Hz, 1 H, 6_a-H), 3.09 (dd, $^2J_{6b,6a}$ = 13.5 Hz, $^3J_{6b,4}$ = 3.2 Hz, 1 H, 6_b-H), 3.18 (dd, $^2J_{2a',2b'}$ = 17.5 Hz, $^3J_{2a',3'}$ = 3.2 Hz, 1 H, 2_a'-H), 3.32 (dd, $^2J_{2b',2a'}$ = 17.5 Hz, $^3J_{2b',3'}$ = 9.1 Hz, 1 H, 2_b'-H), 3.58 (s, 3 H, OMe), 4.14 (dd, $^2J_{5a,5b}$ = 6.3 Hz, $^3J_{5a,4}$ = 2.8 Hz, 1 H, 5_a-H), 4.20 (dd, $^3J_{5b,4}$ = 8.5 Hz, $^2J_{5b,5a}$= 6.3 Hz, 1 H, 5_b-H), 4.59 (dddd, $^3J_{4,6a}$ = 9.6 Hz, $^3J_{4,5b}$ = 8.5 Hz, $^3J_{4,6b}$ = 3.2 Hz, $^3J_{4,5a}$ = 2.8 Hz, 1 H, 4-H), 5.58 (dddd, $^3J_{3',2b'}$ = 9.1 Hz, $^3J_{3',4a'}$ = 6.29 Hz, $^3J_{3',4b'}$ = 6.1 Hz, $^3J_{3',2a'}$ = 3.2 Hz, 1 H, 3'-H), 7.11-7.58 (m, 10 H, arom-H).

Analogue to the literature[12] the assignment of the absolute configuration was done by $\Delta\delta^{RS}$ sign distribution resulting in an (*S*)-configuration of the used alcohol **3a**.

Δδ (=δ_S-δ_R) data for the (*S*)- and (*R*)-MTPA esters (*S*)-7 and (*R*)-7.

#	δ^S	δ^R	$\Delta\delta^{SR}$
5‘	0,86	1,00	<0
4‘	1,74	1,82	<0
3‘	-	-	-
2a‘	3,27	3,18	>0
2b‘	3,31	3,32	<0
5a	4,17	4,14	>0
5b	4,24	4,20	>0
4	4,63	4,59	>0
6a	2,73	2,60	>0
6b	3,24	3,09	>0

(4*R*,3'*S*)-4-Benzyl-3-{3-[(tert-butyldimethylsilyl)oxy]pentanoyl}-oxazolidin-2-one (**4a**)

728 mg (2.63 mmol) compound **3a** were solved in 10 ml dry CH_2Cl_2 and cooled to 0 °C. Freshly distilled 2,6-lutidine (0.64 ml, 0.01 mol, 2.04 eq.) and TBSOTf (0.81 ml, 3.52 mmol, 1.3 eq.) were added dropwise. The reaction was stirred for 1 h at 0 °C, warmed up to room temperature and stirred for another 2 h until full conversion (TLC, petroleum ether:ethyl acetate 80:20). The reaction was diluted with Et_2O, washed with saturated NH_4Cl and saturated $NaHCO_3$ and dried over $MgSO_4$. After evaporation of the solvent the crude product was purified by column chromatography (petroleum ether:ethyl acetate 95:5→90:10). 1.01 g (2.59 mmol, 99%) of the product **4a** was isolated as a clear oil.

TLC: R_f = 0.31 (petroleum ether:ethyl acetate 90:10).

GC-MS (EI, positive-Ion, 70 eV): t_R = 13.67 min, m/z (%) 334 (100) [(M-tBu)$^+$], 276 (80) [(M-TBDMS)$^+$].

Elemental analysis (%) calculated for $C_{21}H_{33}NO_4Si$: C 64.41, H 8.49, N 3.58, found: C 64.01 ± 0.16, H 8.76 ± 0.05, N 3.44 ± 0.02.

IR (Film): $\tilde{v}$[cm^{-1}] = 2957, 2929, 2856, 1782, 1701, 1497, 1463, 1383, 1350, 1302, 1251, 1197, 1099, 1078, 1030, 1005, 938, 834, 775, 748, 701, 663.

$[\alpha]_D^{20}$ = -26.4 (c = 1.0, $CHCl_3$).

^{1}H NMR (600 MHz, $CDCl_3$): δ (ppm) = 0.07 (s, 3 H, $SiMe_a$), 0.09 (s, 3 H, $SiMe_b$), 0.88 (s, 9 H, $SiC(CH_3)_3$), 0.93 (t, $^3J_{5',4'}$ = 7.4 Hz, 3 H, 5'-H), 1.51-1.63 (m, 2 H, 4'-H), 2.76 (dd, $^2J_{6a,6b}$ = 13.4 Hz, $^3J_{6a,4}$ = 9.6 Hz, 1 H, 6-H_a), 3.06 (dd, $^2J_{2'a,2'b}$ = 16.0 Hz, $^3J_{2'a,3'}$ = 5.0 Hz, 1 H, 2'-H_a), 3.15 (dd, $^2J_{2'b,2'a}$ = 16.0 Hz, $^3J_{2'b,3'}$ = 7.2 Hz, 1 H, 2'-H_b), 3.31 (dd, $^2J_{6b,6a}$ = 13.4 Hz, $^3J_{6b,4}$ = 3.3 Hz, 1 H, 6-H_b), 4.14-4.20 (m, 2 H, 5-H), 4.24 (dddd, $^3J_{3',2'b}$ = 7.2 Hz, $^3J_{3',4'}$ = 5.5 Hz, $^3J_{3',4'}$ = 5.4 Hz, $^3J_{3',2'a}$ = 5.0 Hz, 1 H, 3'-H), 4.66 (dddd, $^3J_{4,6a}$ = 9.6 Hz, $^3J_{4,5}$ = 7.0 Hz, $^3J_{4,6b}$ = 3.3 Hz, $^3J_{4,5}$ = 3.3 Hz, 1 H, 4-H), 7.19-7.33 (m, 5 H, arom-H).

^{13}C NMR (151 MHz, $CDCl_3$): δ (ppm) = -4.6 ($SiMe_b$), -4.7 ($SiMe_a$), 9.2 (C-5'), 18.1 (SiC), 25.9 ($SiC(CH_3)_3$), 30.3 (C-4'), 37.9 (C-6), 42.7 (C-2'), 55.2 (C-4), 66.0 (C-5), 69.5 (C-3'), 127.3/129.0/129.4 (Ph), 135.3 (C-7), 153.5 (C-2), 173.0 (C-1').

(4*R*,3'*R*)-4-Benzyl-3-{3-[(tert-butyldimethylsilyl)oxy]pentanoyl}-oxazolidin-2-one (**4b**)

215 mg (0.78 mmol) compound **3b** were solved in 5 ml dry dichloromethane and cooled to 0 °C. Freshly distilled 2,6-lutidine (0.2 ml, 1.72 mol, 2.22 eq.) and TBSOTf (0.25 ml, 1.09 mmol, 1.4 eq.) were added slowly. The reaction was stirred for 1 h at 0 °C, followed by 2 h at room temperature.

After full conversion (reaction control with TLC, petroleum ether:ethyl acetate 80:20) the reaction was diluted with Et_2O, washed with saturated NH_4Cl and saturated $NaHCO_3$-Lösung, respectively. The washed extracts were dried over $MgSO_4$ and the solvent was evaporated. The residue was purified by column chromatography (petroleum ether:ethyl acetate 95:5→90:10) yielded in 285 mg (0.73 mmol, 94%) of the product **4b** as a colorless oil.

TLC: R_f = 0.42 (petroleum ether:ethyl acetate 90:10).

HRMS (ESI, positive-Ion): m/z (%) calculated for $C_{21}H_{34}NO_4Si$ [M +H^+]: 392.22124, found 392.22512.

IR (Film): $\tilde{\nu}$[cm^{-1}] = 2957, 2929, 2856, 2358, 1782, 1699, 1463, 1382, 1351, 1251, 1195, 1116, 1099, 1078, 1029, 1005, 938, 834, 775, 747, 7001.

$[\alpha]_D^{20}$ = -62 (c = 1.0, $CHCl_3$).

^{1}H NMR (600 MHz, $CDCl_3$): δ (ppm) = 0.05 (s, 3 H, $SiMe_a$), 0.08 (s, 3 H, $SiMe_b$), 0.86 [s, 9 H, $SiC(CH_3)_3$], 0.94 (t, $^3J_{5',4'}$ = 7.4 Hz, 3 H, 5'-H), 1.54-1.64 (m, 2 H, 4'-H), 2.74 (dd, $^2J_{6a,6b}$ = 13.4 Hz, $^3J_{6a,4}$ = 9.8 Hz, 1 H, 6-H_a), 2.92 (dd, $^2J_{2'a,2'b}$ = 15.7 Hz, $^3J_{2'a,3'}$ = 4.8 Hz, 1 H, 2'-H_a), 3.33 (dd, $^2J_{6b,6a}$ = 13.4 Hz, $^3J_{6b,4}$ = 3.5 Hz, 1 H, 6-H_b), 3.34 (dd, $^2J_{2'b,2'a}$ = 15.7 Hz, $^3J_{2'b,3'}$ = 7.6 Hz, 1 H, 2'-H_b), 4.16 (m_c, 2 H, 5-H), 4.25 (dddd, $^3J_{3',2'b}$ = 7.6 Hz, $^3J_{3',4'}$ = 5.5 Hz, $^3J_{3',4'}$ = 5.4 Hz, $^3J_{3',2'a}$ = 4.8 Hz, 1 H, 3'-H), 4.65 (dddd, $^3J_{4,6a}$ = 9.8 Hz, $^3J_{4,5}$ = 6.3 Hz, $^3J_{4,6b}$ = 3.6 Hz, $^3J_{4,5}$ = 3.5 Hz, 1 H, 4-H), 7.19-7.33 (m, 5 H, arom.-H).

^{13}C NMR (151 MHz, $CDCl_3$): δ (ppm) = -4.5 ($SiMe_b$), -4.7 ($SiMe_a$), 9.3 (C-5'), 18.0 (SiC), 25.8 ($SiC(CH_3)_3$), 30.4 (C-4'), 38.0 (C-6), 42.0 (C-2'), 55.3 (C-4), 66.0 (C-5), 70.3 (C-3'), 127.3/129.0/129.4 (Ph), 135.4 (C-7), 153.3 (C-2), 171.6 (C-1').

(*S*)-1-{3-[(tert-Butyldimethylsilyl)oxy]pentylidene}-2-(2,4-dinitrophenyl)hydrazine (**5a**)

To a solution of 935 mg (2.3 mmol) of compound **4a** in 40 ml dry dichloromethane at -78 °C were added 10 ml diisobutylaluminium hydride (10 mmol, 1 M in *n*-heptane, 4.35 eq.). The reaction was stirred for 3 h until full conversion (determined with TLC, petroleum ether:ethyl acetate 90:10). The reaction was warmed up to room temperature and quenched with saturated Na-Ka-tartrate solution, yielding a white suspension. The suspension was extracted with diethylether and the solvent was evaporated. The crude product was solved in dry dichloromethane (40 ml) and DNPH (0.68 g, 3.45 mmol, 1.5 eq.) and PTSA*H_2O (200 mg, 1.05 mmol, 0.4 eq.) were added. The red suspension was stirred at room temperature for 1 h, filtrated and washed with *n*-pentane. The solvent was evaporated and the crude product was purified with column chromatography (petroleum ether:ethyl acetate 95:5) yielding product **5a** (489 mg, 1.23 mmol, 52%).

TLC: R_f = 0.48 (petroleum ether:ethyl acetate 90:10).

MS (ESI, positive-Ion): m/z (%) = 435.4 (100) [$(M + K)^+$].

Elemental analysis (%) calculated for $C_{17}H_{28}N_4O_5Si$: C 51.49, H 7.12, N 14.13, found: C 51.48 ± 0.04, H 7.06 ± 0.01, N 13.81 ± 0.03.

Melting point: 83 °C.

IR (Film): $\tilde{\nu}$[cm^{-1}] = 3443, 3306, 3012, 2970, 2850, 1737, 1615, 1515, 1435, 1365, 1325, 1228, 1217, 1203, 1093, 917, 833, 768, 741.

$[\alpha]_D^{20}$ = -61.2 (c = 0.9, $CHCl_3$).

^{1}H NMR (600 MHz, $CDCl_3$): δ (ppm) = 0.06 (s, 3 H, $SiMe_a$), 0.08 (s, 3 H, $SiMe_b$), 0.89 (s, 9 H, $SiC(CH_3)_3$), 0.94 (t, $^3J_{5,4}$ = 7.44 Hz, 3 H, 5'-H), 1.47 (m_c, 2 H, 4'-H), 2.55 (ddd, $^2J_{2a,2b}$ = 14.3 Hz, $^3J_{2a,1}$ = 6.1 Hz, $^3J_{2a,3}$ =

6.1 Hz, 1 H, 2'-H_a), 2.60 (ddd, $^2J_{2b,2a}$ = 14.3 Hz, $^3J_{2a,1}$ = 5.3 Hz, $^3J_{2a,1}$ = 4.8 Hz, 1 H, 2'-H_b), 3.91 (dddd, $^3J_{3,2a}$ = 6.1 Hz, $^3J_{3,4}$ = 6.1 Hz, $^3J_{3,4}$ = 6.0 Hz, $^3J_{3,2b}$ = 4.8 Hz, 1 H, 3'-H), 7.55 (dd, $^3J_{1,2a}$ = 6.1 Hz, $^3J_{1,2b}$ = 5.3 Hz, 1 H, 1'-H), 7.93 (d, $^3J_{6',5'}$ = 9.6 Hz, 1 H, 6''-H), 8.30 (dd, $^3J_{5',6'}$ = 9.6 Hz, $^3J_{5',3'}$ = 2.6 Hz, 1 H, 5''-H), 9.12 (d, $^3J_{3',5'}$ = 2.6 Hz, 1 H, 3''-H), 11.04 (s, 1 H, 2-H).

^{13}C NMR (151 MHz, $CDCl_3$): δ (ppm) = -4.5 ($SiMe_b$), -4.6 ($SiMe_a$), 9.7 (C-5'), 18.1 (SiC), 25.8 ($SiC(CH_3)_3$), 30.2 (C-4'), 39.5 (C-2'), 71.7 (C-3'), 116.5 (C-6''), 124.0 (C-3''), 128.8 (C-2''), 130.0 (C-5''), 137.9 (C-4''), 145.1 (C-1''), 150.5 (C-1').

(*R*)-1-{3-[(tert-Butyldimethylsilyl)oxy]pentylidene}-2-(2,4-dinitrophenyl)hydrazine (**5b**)

To a solution of 181 mg (0.51 mmol) of compound **4b** in 10 ml dry dichloromethane at -78 °C diisobutylaluminium hydride (2 ml, 2.5 mmol, 1 M in *n*-hexane, 4.89 eq.) was added. The reaction was stirred for 3 h until full conversion (TLC, petroleum ether:ethyl acetate 90:10). The reaction was warmed up to room temperature and quenched with saturated Na-Ka-tartrate solution (white suspension). The suspension was extracted with diethylether and the solvent was evaporated. The crude product was solved in dry dichloromethane (10 ml) and DNPH (0.15 g, 0.77 mmol, 1.5 eq.) and PTSA*H_2O (70 mg, 0.41 mmol, 0.87 eq.) were added. The red suspension was stirred at room temperature for 1 h, filtrated and washed with *n*-pentane. The solvent was evaporated and the crude product was purified with column chromatography (petroleum ether:ethyl acetate 95:5) yielding the product **5b** (104 mg, 0.26 mmol, 56%).

The analytical data were found to be identical with those from **5a**.

$[\alpha]_D^{20}$ = +69.5 (c = 1.1, $CHCl_3$).

(*S,E*)-1-[2-(2,4-Dinitrophenyl)hydrazono]pentan-3-ol (**6a**)

A solution of compound **5a** (489.0 mg, 1.23 mmol) in 15 ml dry tetrahydrofuran was cooled to 0 °C. Hydrogen fluoride pyridine (1.5 ml, 11.64 mmol, 70%, 9.45 eq.) was added. The reaction was stirred for 6 h at 0 °C and for 16 h at 6 °C, diluted with ethyl acetate and washed with saturated $NaHCO_3$-solution. The solvent was evaporated and purified by means of column chromatography (petroleum ether:ethyl acetate 80:20) and recrystallization from *n*-pentane yielded 122 mg (0.43 mmol, 35%) of product **6a**.

TLC: R_f = 0.11 (petroleum ether:ethyl acetate 80:20).

HRMS (ESI, positive-Ion): m/z (%) calculated for $C_{11}H_{15}N_4O_5$ [M +H]: 283.09977, found: 283.10366.

Elemental analysis (%) calculated for $C_{11}H_{14}N_4O_5$: C 46.81, H 5.00, N 19.85, found: C 46.89 ± 0.07, H 4.92 ± 0.01, N 19.40 ± 0.05.

Melting point: 99.5 °C.

IR (Film): $\tilde{\nu}$[cm^{-1}] = 3290, 3108, 2969, 2920, 1737, 1614, 1587, 1509, 1417, 1375, 1305, 1262, 1217, 1136, 1073, 1037, 1017, 975, 939, 922, 831, 741, 724, 686, 660.

$[\alpha]_D^{20}$= 22.4 (c = 1.2, $CHCl_3$).

HPLC (Chrialpak IA, 0.5 ml*min^{-1}, 80:20 *n*-heptane/2-propanol, 345 nm: **6a** t_R= 79.0.

^{1}H NMR (600 MHz, $CDCl_3$): δ (ppm) = 1.02 (t, $^3J_{5,4}$ = 7.5 Hz, 3 H, 5'-H), 1.59-1.64 (m, 2 H, 4'-H), 1.95 (d, $^3J_{OH,3}$ = 4.3 Hz, 1 H, OH), 2.55 (ddd, $^2J_{2a,2b}$ = 15.5 Hz, $^3J_{2a,3}$ = 8.3 Hz, $^3J_{2a,1}$ = 5.3 Hz, 1 H, 2'-H_a), 2.65 (ddd, $^2J_{2b,2a}$ = 15.5 Hz, $^3J_{2a,3}$ = 3.6 Hz, $^3J_{2a,1}$ = 5.3 Hz, 1 H, 2'-H_b), 3.95 (m_c, 1 H, 3'-H), 7.66 (t, $^3J_{1,2a}$ = 5.3 Hz,

$^{3}J_{1,2b}$ = 5.3 Hz, 1 H, 1'-H), 7.90 (d, $^{3}J_{6',5'}$ = 9.5 Hz, 1 H, 6''-H), 8.30 (dd, $^{3}J_{5',6'}$ = 9.5 Hz, $^{3}J_{5',3'}$ = 2.6 Hz, 1 H, 5''-H), 9.11 (d, $^{3}J_{3',5'}$ = 2.6 Hz, 1 H, 3''-H), 11.08 (s, 1 H, 2-H).

^{13}C NMR (151 MHz, $CDCl_3$): δ (ppm) = 9.8 (C-5'), 30.3 (C-4'), 39.5 (C-2'), 70.9 (C-3'), 116.4 (C-6''), 123.5 (C-3''), 129.0 (C-2''), 130.0 (C-5''), 138.0 (C-4''), 144.9 (C-1''), 150.3 (C-1').

(*R*,*E*)-1-[2-(2,4-Dinitrophenyl)hydrazono]pentan-3-ol (**6b**)

A solution of compound **5b** (45 mg, 0.13 mmol) in 2 ml dry THF was cooled to 0 °C. Hydrogen fluoride pyridine (0.2 ml, 0.78 mmol, 70%, 13.7 eq.) was added. The reaction was stirred for 2.5 h, diluted with ethyl acetate and washed with saturated $NaHCO_3$-solution. The solvent was evaporated and purified by means of column chromatography (petroleum ether:ethyl acetate 80:20) and recrystallization from *n*-pentane yielded 25 mg (0.09 mmol, 78%) of product **6b**.

The analytical data were found to be identical with those from **6a**.

HPLC (Chrialpak IA, 0.5 ml*min^{-1}, 80:20 *n*-heptane/2-propanol, 345 nm: **6b** t_R = 70.9.

$[\alpha]_D^{20}$ = -20.3 (c = 0.8, $CHCl_3$).

Supplementary References

1. Jacobs, D.J., Rader, A.J., Kuhn, L.A. & Thorpe, M.F. Protein flexibility predictions using graph theory. *Proteins.* **44**, 150-165 (2001).
2. Jacobs, D.J. & Thorpe, M.F. Generic rigidity percolation: The pebble game. *Phys. Rev. Lett.* **75**, 4051-4054 (1995).
3. Jacobs, D.J. & Hendrickson, B. An algorithm for two-dimensional rigidity percolation: The pebble game. *J. Comput. Phys.* **137**, 346-365 (1997).
4. Radestock, S. & Gohlke, H. Exploiting the Link between Protein Rigidity and Thermostability for Data-Driven Protein Engineering. *Eng. Life Sci.* **8**, 507-522 (2008).
5. Rathi, P.C., Radestock, S. & Gohlke, H. Thermostabilizing mutations preferentially occur at structural weak spots with a high mutation ratio. *J. Biotechnol.* **159**, 135-144 (2012).
6. Rader, A.J., Hespenheide, B.M., Kuhn, L.A. & Thorpe, M.F. Protein unfolding: rigidity lost. *Proc. Natl. Acad. Sci. USA* **99**, 3540-3545 (2002).
7. Radestock, S. & Gohlke, H. Protein rigidity and thermophilic adaptation. *Proteins: Struct. Funct. Bioinf.* **79**, 1089-1108 (2011).
8. Dahiyat, B.I., Gordon, D.B. & Mayo, S.L. Automated design of the surface positions of protein helices. *Protein Sci.* **6**, 1333-1337 (1997).
9. Pfleger, C., Radestock, S., Schmidt, E. & Gohlke, H. Global and Local Indices for Characterizing Biomolecular Flexibility and Rigidity. *J. Comput. Chem.* **34**, 220-233 (2013).
10. Rathi, P.C., Jaeger, K.E. & Gohlke, H. Structural rigidity and protein thermostability in variants of lipase A from Bacillus subtilis. *PLOS One*, **10**(7) e0130289 (2015).
11. Ager, D.J., Allen, D.R. & Schaad, D.R. Simple and Efficient N-Acylation Reactions of Chiral Oxazolidine Auxiliaries. *Synthesis* **1996**, 1283-1285 (1996).
12. Seco, J.M., Quiñoá, E. & Riguera, R. Assignment of the Absolute Configuration of Polyfunctional Compounds by NMR Using Chiral Derivatizing Agents. *Chem. Rev.* **112**, 4603-4641 (2012).
